# Multiscale Understanding of Covalently Fixed Sulfur–Polyacrylonitrile Composite as Advanced Cathode for Metal–Sulfur Batteries

**DOI:** 10.1002/advs.202101123

**Published:** 2021-08-08

**Authors:** Mohammad Shamsuddin Ahmed, Suyeong Lee, Marco Agostini, Min‐Gi Jeong, Hun‐Gi Jung, Jun Ming, Yang‐Kook Sun, Jaekook Kim, Jang‐Yeon Hwang

**Affiliations:** ^1^ Department of Materials Science and Engineering Chonnam National University Gwangju 61186 Republic of Korea; ^2^ Department of Physics Chalmers University of Technology Göteborg SE41296 Sweden; ^3^ Center for Energy Storage Research Clean Energy Institute Korea Institute of Science and Technology Seoul 02792 Republic of Korea; ^4^ State Key Laboratory of Rare Earth Resource Utilization Changchun Institute of Applied Chemistry CAS Changchun 130022 China; ^5^ Department of Energy Engineering Hanyang University Seoul 04763 Republic of Korea

**Keywords:** chemical structure, metal–sulfur batteries, sulfurized polyacrylonitrile, universal cathodes

## Abstract

Metal–sulfur batteries (MSBs) provide high specific capacity due to the reversible redox mechanism based on conversion reaction that makes this battery a more promising candidate for next‐generation energy storage systems. Recently, along with elemental sulfur (S_8_), sulfurized polyacrylonitrile (SPAN), in which active sulfur moieties are covalently bounded to carbon backbone, has received significant attention as an electrode material. Importantly, SPAN can serve as a universal cathode with minimized metal–polysulfide dissolution because sulfur is immobilized through covalent bonding at the carbon backbone. Considering these unique structural features, SPAN represents a new approach beyond elemental S_8_ for MSBs. However, the development of SPAN electrodes is in its infancy stage compared to conventional S_8_ cathodes because several issues such as chemical structure, attached sulfur chain lengths, and over‐capacity in the first cycle remain unresolved. In addition, physical, chemical, or specific treatments are required for tuning intrinsic properties such as sulfur loading, porosity, and conductivity, which have a pivotal role in improving battery performance. This review discusses the fundamental and technological discussions on SPAN synthesis, physicochemical properties, and electrochemical performance in MSBs. Further, the essential guidance will provide research directions on SPAN electrodes for potential and industrial applications of MSBs.

## Graphical abstract

Sulfurized polyacrylonitrile (SPAN), as a universal sulfur cathode, in which active sulfur moieties are bound to a carbon backbone that can allow all sulfur atoms to become electrochemically active in metal–sulfur batteries. This review covers the key technological developments for the synthesis, physicochemical properties, and electrochemical performances of SPAN.

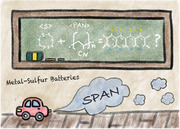

## 1 Introduction

The environmental impacts and risks of natural resource depletion associated with the immense consumption of traditional fossil fuels have stimulated a global demand for renewable and clean energy such as, solar, wind, and tidal.^[^ ^1^, ^2^ ^]^ However, due to their irregular natures, such renewable energies need to be stored so that they are available when needed. Rechargeable batteries are considered the most promising storage and conversion systems because of the high possibility of modulating the shape and having good energy density.^[^ ^2^ ^]^ Lithium‐ion batteries (LIBs), among others, are well‐established and abundantly used in the portable electronics market. Moreover, they have demonstrated a suitable system in the electric vehicles (EVs) market. However, the energy density delivered by commercial LIBs is limited by the electrochemical mechanism, that is, the intercalation of Li^+^ in both anode and cathode to a low value is not enough for large‐scale applications, such as, stationary storage. Additional problems related to the abundance and high cost of active materials, as well as, safety concerns, necessitate the development of alternative battery systems.^[^ ^2^, ^3^, ^4^ ^]^

**Figure 1:**
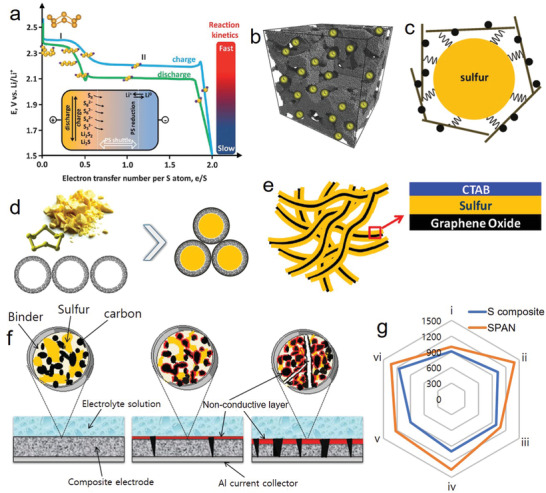
Typical electrochemical charge/discharge voltage profile of sulfur versus Li^+^. Inset: a) Polysulfide (PS) shuttle. Reproduced with permission.^[^ ^52^ ^]^ Copyright 2013, RoyalSociety of Chemistry. b) Encapsulation of sulfur particles into the porous carbon. Reproduced with permission.^[^ ^21^ ^]^ Copyright 2015, Wiley‐VCH. c) Carbonized interlayer system for intercepting the migrating polysulfide species. Reproduced with permission.^[^ ^23^ ^]^ Copyright 2011, American Chemical Society. d) Impregnation of sulfur in carbon matrix through core‐shell engineering. Reproduced with permission.^[^ ^22^ ^]^ Copyright 2014, American Chemical Society. e) A layer‐by‐layer approach of sulfur immobilized with GO.^[^ ^24^ ^]^ Copyright 2013, American Chemical Society. f) Schematic structure of the sulfur cathode, before and after polysulfide dissolution. Reproduced with permission.^[^ ^21^ ^]^ Copyright 2015, American Chemical Society. g) Comparison of stable discharge specific capacity (mAh g^−1^) produced from SPAN (i) CNF‐S,^[^ ^18^ ^]^ ii) SPAN/CNT‐12,^[^ ^44^ ^]^ iii) *c*‐PANS,^[^ ^45^ ^]^ iv) S‐a‐MCNF,^[^ ^46^ ^]^ v) Se_0.06_SPAN,^[^ ^47^ ^]^ vi) Te_0.04_S_0.96_@pPAN^[^ ^48^ ^]^) and sulfur composite (i) NOCC@S_8_/rGO,^[^ ^49^ ^]^ ii) S@PC 12h,^[^ ^50^ ^]^ iii) S/GO,^[^ ^51^ ^]^ iv) Li2S/N,P–C,^[^ ^52^ ^]^ v) S@MWCNT/PAA,^[^ ^53^ ^]^ vi) TiN@NG/S^[^ ^54^ ^]^) cathodes in 1 m LiTFSI in DOL/DME containing with LiNO_3_.

Metal–sulfur batteries (MSBs) have shown high potential because of their particular electrochemical mechanism of conversion reaction, which rises the theoretical capacity to 5–8 times higher than that provided by the intercalation chemistry of LIBs. Since 1962,^[^ ^5^, ^6^ ^]^ sulfur has been explored as a cathodic material in metal battery systems. Sulfur is abundant (being 17th among all elements and 7th among non‐metallic elements in nature),^[^ ^7^ ^]^ eco‐friendly, low cost, has high theoretical energy density (2600 Wh kg^−1^ vs Li^+^ reaction), and high theoretical capacity (1675 mAh g^−1^ vs Li^+^ reaction).^[^ ^5^, ^8^, ^9^, ^10^, ^11^, ^12^ ^]^ Moreover, it is compatible as a cathode with a series of metal through the conversion reaction forming metal–sulfide such as, Li_2_S, Na_2_S, MgS, and AlS*_x_*.^[^ ^13^, ^14^, ^15^ ^]^ However, MSBs have continued to fail in attracting significant attention because they experience several problems that originate from sulfur chemistry. First, sulfur has a very low electronic conductivity (5 × 10^−30^ S cm^−1^),^[^ ^16^, ^17^, ^18^ ^]^ which makes it impossible to use as elemental element in batteries. A second major problem is related to the redox reaction of sulfur. It relies on the systematic formation of lithium‐polysulfides (LiPs) with different lengths that easily diffuse from the cathode to the anode through a concentration gradient (**Figure** **1**a). The dissolution of sulfur in the electrolyte as LiP intermediates, particularly long‐chain, results in active material loss. This triggers undesirable shuttle reactions, leading to fast capacity decay, low coulombic efficiency (CE), and high self‐discharge rate.^[^ ^18^ ^]^

To overcome the aforementioned problems, different approaches have been proposed. Among them, the most common approaches for improving the sulfur performance in batteries are i) the synthesis of sulfur–carbon composites by encapsulate sulfur in the mesopores of carbon matrixes (Figure 1b),^[^ ^19^, ^20^, ^21^ ^]^ ii) introduction of a porous interlayer between the separator and the sulfur electrodes that mitigates the migration of active material (Figure 1c),^[^ ^23^ ^]^ iii) formation of core‐shell hierarchical porous carbon spheres or tubes containing sulfur (Figure 1d),^[^ ^22^ ^]^ and iv) use of graphene for wrapping the elemental sulfur and enhancing the electronic conductivity (Figure 1e).^[^ ^24^ ^]^ Despite demonstrating an improvement in Li/S cycling performance, these approaches demonstrated structure collapses and slabbing of metal sulfide precipitation on the electrode surface, leading to electronical disconnection from the bulk of the electrode on cycling (Figure 1f).^[^ ^21^ ^]^ Composites based on polymer have also been extensively investigated, in particular, the use of conductive polymers, but with low capacity performance (<260 mAh g^−1^) and poor inherent electronic conductivity of the sulfur/polymer compound.^[^ ^25^, ^26^, ^27^, ^28^ ^]^

A new class of sulfur cathodes has been introduced, with active sulfur moieties chemically bound to an electronically conductive and nonreactive carbon backbone. Wang et al. first reported a sulfurized/carbonized polyacrylonitrile (SPAN) composite material synthesized from polyacrylonitrile (PAN) and sulfur via thermal treatment in 2002.^[^ ^29^, ^30^ ^]^ It demonstrated a specific capacity of approximately 850 mAh g^−1^, with an improved electronic conductivity and unique properties such as better feasibility upon cycling, preventing sulfur from aggregating, and inhibiting the dissolution of the discharge products, thereby allowing all sulfur atoms to be fully electrochemically active. Over the following years, SPAN has become a popular cathode material^[^ ^4^, ^31^, ^32^, ^33^, ^34^, ^35^, ^36^, ^37^, ^38^, ^39^, ^40^ ^]^ owing to have several advantages over other sulfur based cathodes such as, higher conductivity, better compatibility toward carbonate electrolytes, low polysulfide dissolution, and can be used as universal cathode for many MSBs. These features are generally important to achieve a highly reversible specific capacity and cycling stability in sulfur batteries.^[^ ^4^, ^33^, ^41^, ^42^, ^43^ ^]^ An instant comparison of stable discharge specific capacity from arbitrarily chosen SPAN^[^ ^18^, ^44^, ^45^, ^46^, ^47^, ^48^ ^]^ and sulfur (S_8_)‐ and Li_2_S‐composite^[^ ^49^, ^50^, ^51^, ^52^, ^53^, ^54^ ^]^ cathodes tested in a common electrolyte, 1 m LiTFSI in 1,3‐dioxolane/dimethoxyethane (DOL/DME) containing with LiNO_3_ (Figure 1g) which demonstrating the superoprity of SPAN over sulfur composite. However, the low operation voltage and low sulfur content of SPAN still need to improve for its practical use.

**Figure 2:**
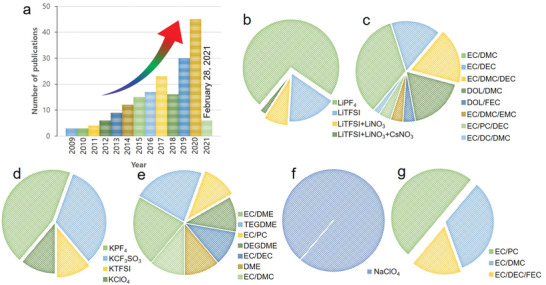
a) Number of publications on SPAN‐based electrodes including all metal‐battery systems, published from 2009 to 2020 (data obtained from Web of Science) and electrolyte summary of Li—S, K—S, and Na—S battery systems; b) salts and c) solvents for Li—S cathodes; d) salts and e) solvents for K—S cathodes; and f) salts and g) solvents for the Na—S cathodes discussed in this review.

A summary of the studies on SPAN electrodes is provided **Figure** **2**. The number of publications on SPAN gradually increased from 2009 to 2020, reflecting a higher research interest (Figure 2a). In addition, the compatibility of both carbonate‐ and ether‐based electrolytes was confirmed by frequently using SPAN cathode (Figure 2b–g), that is, mostly impossible for conventional sulfur cathodes due to sulfur dissolution and electrolytes decomposition.

**Figure 3:**
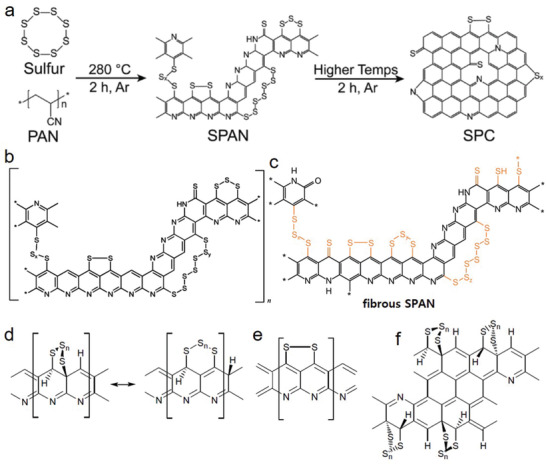
Schematic of covalent chemical structures for SPAN materials with —[S]*_n_*_= 2,…8_— chains. a,b) Reproduced with permission.^[^ ^44^ ^]^ Copyright 2011, American Chemical Society. c) Reproduced with permission.^[^ ^34^ ^]^ Copyright 2017, American Chemical Society. d,e,f) Reproduced with permission.^[^ ^32^ ^]^ Copyright 2014, MDPI.

**Figure 4:**
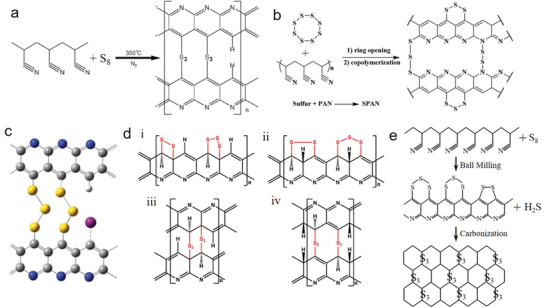
Schematic of covalent structure of some SPAN materials with —[S]*_n_*_≤ 3_— chains. a) Reproduced with permission.^[^ ^58^ ^]^ Copyright 2020, Royal Society of Chemistry. b) Reproduced with permission.^[^ ^77^ ^]^ Copyright 2010, MDPI. c) Reproduced with permission.^[^ ^74^ ^]^ Copyright 2019, Elsevier. d) Reproduced with permission.^[^ ^44^ ^]^ e) Reproduced with permission.^[^ ^33^ ^]^ Copyright 2015, American Chemical Society.

The polymeric chain of PAN becomes cyclized and dehydrogenated by losing H_2_S under heating, resulting to sulfurized carbon double bonds. Previous studies have described the structural features, reaction mechanisms, and optimization of synthesis conditions and characterization of SPAN.^[^ ^32^ ^]^ Most studies reported that sulfur can be covalently bonded onto the carbon backbone by forming —[S]*_n_*_= 2,…8_— chains (**Figure** **3**)^[^ ^32^, ^34^, ^55^, ^56^, ^57^ ^]^ where the molecular formula of SPAN is C_8.6_N_2.2_H_1.2_S_3.3_O*_x_* or C_8.5_N_2.3_HS_3.4_O*_y_* (atomic ratio of C/S is 2.61 or 2.5)^[^ ^34^ ^]^ and the length of sulfur chain is (—[S]*_n_*_≤ 3_—) (**Figure** **4**);^[^ ^33^, ^44^, ^58^ ^]^ where the molecular formula of SPAN is C_4_N_1.3_HS_1.6_ (atomic ratio of C/S 2.5).^[^ ^44^ ^]^

**Figure 5:**
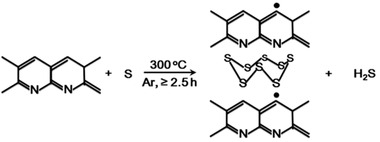
Schematic of noncovalent chemical structures for SPAN composite containing sulfur nanoclusters. Reproduced with permission.^[^ ^59^ ^]^ Copyright 2020, Royal Society of Chemistry.

Researchers have also reported that elemental sulfur is non‐covalently immobilized while being covalently bonded onto the carbon backbones in the form of sulfur nanoclusters (**Figure** **5**),^[^ ^59^ ^]^ in which sulfur rings/particles can be partially trapped between PAN rings, with no structural changes following further thermal treatment. The *π*‐electron of the carbon atoms in PAN rings can contribute to noncovalent immobilization of sulfur.^[^ ^56^ ^]^ When considering the similar structure of sulfur‐polyaniline (SPANI) to SPAN, similar phenomena have been observed in self‐assembled SPANI, whereby sulfur is physically encapsulated into the hollow voids and covalently bonded on the SPANI backbone within the cross‐linked polymer structure.^[^ ^60^, ^61^ ^]^ Therefore, it is highly possible to have noncovalently bonded sulfur nanoclusters in the SPAN structure.

The reversible electrochemical reaction between the SPAN cathode (S_ctd_) and metal anode (M_and_) experiences reduction of SPAN to accept positively charged metal (M*^n^* ^+^) (Equation (1)), as well as, oxidation of metal by releasing electron(s), thereby producing positively charged metals during discharge (Equation (2)).

(1) $$S_{\mathrm{ctd}}+ne^{-}+M^{n+}↔S-M$$

(2) $$M_{\mathrm{and}}↔ne^{-}+M^{n+}$$

Although many possible ways have been explored to prepare SPAN, the chemical structure, sulfur state, and electrochemical properties are not completely understood. Despite several reviews reporting on sulfur cathode batteries,^[^ ^8^, ^16^, ^57^, ^58^, ^59^, ^60^, ^61^, ^62^, ^63^, ^64^, ^65^, ^66^, ^67^, ^68^, ^69^ ^]^ a systematic review focusing on the topics associated with SPAN electrodes is not present in the existing literature. Herein, we summarize the recent developments in the study of SPAN electrodes and provide insight into the fundamental understanding of its electrochemical properties, versatile synthesis processes, and advanced characterization methods.

## 2 Synthesis and Characterization of Sulfurized Polyacrylonitrile

SPAN is an electrochemically active, conductive, and metal‐free sulfur/carbon composite that is used as a cathode in metal‐battery systems. Since its first report in 2002, numerous synthesis methods have been explored for preparing composites using polymers, nanocarbon materials, molecules, or metals.^[^ ^57^ ^]^ All methods and composite formations aim to add sulfur moieties within the carbon for improving the electrochemical performance. The performance and physical properties of SPAN materials are strongly related to the synthetic conditions. Therefore, all types of methods and composites and their respective advantages are discussed.

### 2.1 Reaction Mechanism of Thermal Synthesis of Sulfurized Polyacrylonitrile

**Figure 6:**
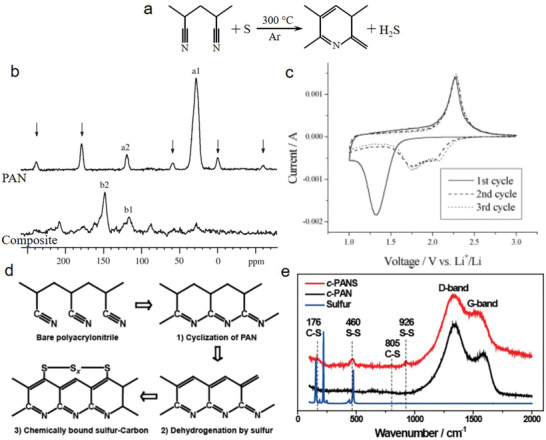
a) PAN side chain to cyclical through reaction with sulfur, b) ^13^C NMR spectra of pure PAN and composite of sulfur and PAN, c) cyclic voltammogram of composite with 49% sulfur at a scan rate of 0.05 mV s^−1^. Reproduced with permission.^[^ ^30^ ^]^ Copyright 2017, Wiely‐VCH. d) Structural changes during carbonization and sulfurization of *c*‐PANS and e) Raman spectra of *c‐*PANS and *c‐*PAN. Reproduced with permission.^[^ ^31^ ^]^ Copyright 2013, American Chemical Society.

The first SPAN composite reported in the literature was prepared via thermal treatment process, a key reaction for the preparation of SPAN materials.^[^ ^29^, ^30^ ^]^ In the presence of elemental sulfur at 300 °C, PAN side chains become cyclic through the dehydrogenation reaction with sulfur (**Figure** **6**a). According to the ^13^C nuclear magnetic resonance (NMR) (Figure 6b), the downshift of peak a1 to b1 indicated that sp^3^ carbon bonds changed to sp^2^ carbons in PAN. The downshift of peak a2 to b2 indicated the cyclization of the —CN group. Moreover, Fourier‐transform infrared spectroscopy (FT‐IR) results supported the ^13^C NMR by detecting the presence of new peaks at 1498 and 1427 cm^−1^ for the C = C bond and aromatic cyclic structure, respectively. The prepared composite showed the characteristic anodic and cathodic peaks in the cyclic voltammetry test (Figure 6c) and a stable charge‐discharge profile with a specific capacity up to 850 mAh g^−1^ at the first cycle. This capacity was maintained after 50 cycles, which indicates a 95.2% sulfur utilization, according to the reaction 2Li + S = Li_2_S.

Hwang et al.^[^ ^31^ ^]^ prepared a SPAN electrode employing a different strategy. First, 1D PAN nanofibers (NFs) were prepared by electrospinning technique to maintain a homogenous diameter. Subsequently, the prepared 160‐nm diameter PAN NFs were mixed with elemental sulfur powder in a mass ratio of 20:80 and heated at 450 °C for 6 h under nitrogen flow. The probable reaction mechanism of the mixture was explained to follow three major chemical reactions (Figure 6d). The cyclization of PAN results from the bonding of the cleaved nitrogen to the carbon at a certain temperature.^[^ ^9^, ^30^ ^]^ With increasing temperature, the dehydrogenation reaction occurs with sublimed sulfur, resulting in *π*‐conjugated main chains while producing H_2_S.^[^ ^37^, ^70^ ^]^ Finally, at elevated temperatures, elemental sulfur changes to sulfur free radicals while forming the final C—S bond with the PAN‐derived carbon matrix or sulfur nanodomains, consisting of sulfur atom chains of various lengths.^[^ ^44^, ^71^ ^]^ These atomic arrangements were confirmed by Raman analysis (Figure 6e), whose results supported the mechanism discussed above. The cycling performance of *c*‐PANS NFs at various C‐rates and discharge–charge capacities were the same in each cycle. Many other SPAN materials have been prepared with the same strategy.^[^ ^38^, ^72^, ^73^, ^74^, ^75^, ^76^ ^]^

**Figure 7:**
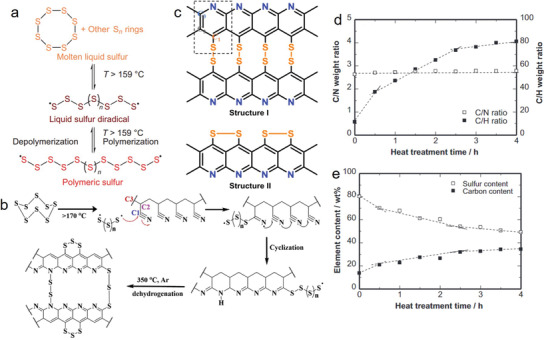
a) Schematic for thermal ring‐opening polymerization of S_8_ to polymeric sulfur diradical chain ends. Reproduced with permission.^[^ ^79^ ^]^ Copyright 2014, Elsevier B.V. b) Proposed reaction mechanism for the synthesis of SPAN initiated by polysulfane diradical chains during thermal treatment. Reproduced with permission.^[^ ^77^ ^]^ Copyright 2010, MDPI. c) Proposed molecular structure of SPAN. Reproduced with permission.^[^ ^78^ ^]^ Copyright 2018, Elsevier B.V. d) Effect of thermal treatment duration on C/N and C/H weight ratios, and e) S and C contents of the S/DPAN composites. Reproduced with permission.^[^ ^59^ ^]^ Copyright 2020, Royal Society of Chemistry.

It is crucial to understand the C—S bond formation between the elemental sulfur and PAN‐derived carbon backbone via covalent reaction.^[^ ^77^, ^78^ ^]^ It is established that elemental sulfur exists in the form of an eight‐membered ring (cyclooctasulfur, S_8_) at normal conditions and melts (liquid phase) at ≈124 °C. The liquid sulfur transforms into linear polysulfane through the ring‐opening polymerization of the S_8_ monomer in an equilibrium state with diradical chain ends above 159 °C. Subsequently, linear polysulfane polymerizes to polymeric sulfur with the remaining diradical chain ends (**Figure** **7**a).^[^ ^79^ ^]^ The diradical chain of polymeric sulfur initiates the formation of the heteroatomic structure by attacking the nitrile carbon (C1), thereby forming C—S bonds and causing sulfurization of the non‐cycled PAN. Subsequently, at the elevated temperature, the sulfurized non‐cycled PAN becomes cyclized, yielding a conjugated heteroaromatic polypyridine ring structure containing C = C and C = N bonds, followed by a hydrogenation reaction. This indicates that both heteroatoms (N and S) are attached at the same side of the polypyridine ring unit (Figure 7b).^[^ ^34^, ^80^ ^]^ However, some studies have reported that PAN is cyclized first at an elevated temperature, and then sulfur radical chains combine chemically with the positively polarized carbon atoms in the polypyridine rings forming C—S bonds,^[^ ^81^ ^]^ along with the hydrogenation reaction at high temperature. The position of heteroatoms, including carbon atoms, in the structural unit are proposed (dotted line) by Wang et al.^[^ ^78^ ^]^ (Figure 7c), where both heteroatoms are attached at the opposite side of the polypyridine ring unit. The other part of the sulfur chain is bonded through S—S bonds between two sulfur radical chains of adjacent polypyridine rings (Figure 7c). Therefore, the loss of hydrogen and sulfur with the increasing wt% of carbon due to the time‐dependent thermal treatment (dehydrogenation) caused by the H_2_S production was identified (Figure 7d,e).

### 2.2 Methods of Sulfurized Polyacrylonitrile Synthesis

As SPAN is considered to be a promising cathode material for metal‐batteries, many researchers are attempting to manufacture them in different ways to improve the electrochemical performance and stability. Their strategy is divided into three categories: i) chemical approach, ii) physical approach, and iii) other approaches.

#### 2.2.1 Pre‐Treatment for Polyacrylonitrile Preparation to Sulfurized Polyacrylonitrile Synthesis: Chemical Approach

**Figure 8:**
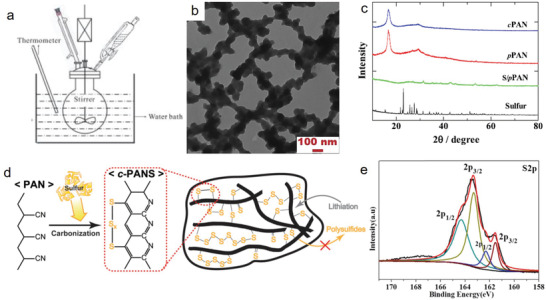
a) Schematic of the equipment for preparing suspension polymer. Reproduced with permission.^[^ ^86^ ^]^ Copyright 2015, Royal Society of Chemistry. b) TEM image of network‐like S/*_p_* PAN composite. c) XRD patterns of *_c_* PAN, *_p_* PAN, S/*_p_* PAN composite, and sulfur precursor. Reproduced with permission.^[^ ^88^ ^]^ Copyright 1979, Academic Press. d) Schematic of synthetic and molecular structure of SPAN (*c*‐PANS). Reproduced with permission.^[^ ^45^ ^]^ Copyright 2004, Elsevier B.V. e) Representative core level of S 2p XPS spectra. Reproduced with permission.^[^ ^58^ ^]^ Copyright 2020, Royal Society of Chemistry.

Chemical reaction has been introduced as a pre‐treatment for the preparation of SPAN followed by thermal treatment for achieving better electronic conduction. The chemical approach leads to the tuning of the surface morphology and increase in the porosity, thereby resulting in better electrochemical activity.^[^ ^82^, ^83^, ^84^, ^85^ ^]^ Zhu et al.^[^ ^86^, ^87^ ^]^ prepared SPAN using suspension polymerization. For polymerization, the polyvinyl alcohol was dispersed in 100 mL of water at 65 °C, and then, measured amounts of dimethyl formamide (DMF), acrylonitrile (AN), and sublimated sulfur were added sequentially and stirred violently. Finally, azodiisobutyronitrile was added as a reaction promoter and then kept for 3 h for polymerization (**Figure** **8**a). The prepared polymer was carbonized at 300 °C for 8 h. The prepared SPAN showed a particulate morphology with a diameter of ≈3–5 µm at lower magnification, whereas showed a homogenous flake‐like crystalline morphology at higher magnification. Moreover, it showed good cycling properties in lithium batteries. The initial discharge capacity was estimated to be 546.6 mAh g^−1^, with good reversibility in the subsequent cycles. A lower but stable discharge capacity of 416.4 mAh g^−1^ was observed following the second cycle and remained at ≈400 mAh g^−1^ up to the 30th cycle, with 33.41% sulfur utilization.

Another SPAN material was prepared via in situ polymerization with a different morphology.^[^ ^88^ ^]^ Through this process, a network‐like SPAN (denoted as S/*_p_* PAN) was prepared with morphology that was different from the conventionally prepared SPAN (denoted as S/*_c_* PAN) (Figure 8b). A measured amount of AN, potassium persulfate, and sodium dodecyl sulfate were mixed into a nano‐sulfur aqueous suspension under stirring at 70 °C for 10 h for in situ polymerization. The suspension was then dried and heated at 350 °C for 6 h under argon gas to melt the sulfur and react with PAN. The polymerization reaction was confirmed by FT‐IR, wherein characteristic peaks at 2244 cm^−1^ revealed the presence of –CN groups, whereas those at 1455 cm^−1^ revealed that of the —CH_2_ group, indicating that PAN was successfully obtained via the chemical polymerization method. Furthermore, the presence of sulfur in SPAN was confirmed by X‐ray diffraction (XRD) patterns (Figure 8c). The comparative XRD showed a typical peak of PAN at 16.8°, which confirmed the crystal structure of prepared *_p_* PAN. The characteristic peaks of elemental sulfur disappeared from the prepared SPAN XRD pattern, indicating that sulfur was embedded as fine particles in the polymer matrix. Benefiting from this unique network‐like structure, the S/*_p_* PAN composite cathode demonstrated enhanced reversibility, resulting in a discharge capacity of 1177 and 981 mAh g^−1^ at the second cycle, with ≈100% of those values retained over 100 cycles at 0.5 C and 1 C rates, respectively. Zhang et al.^[^ ^89^ ^]^ prepared an S/cPAN/carbon multi‐composite based on the dual mode of fixing sulfur onto the PAN matrix and activated carbon via chemical bonding and physical loading, respectively, which was beneficial for the high loading of sulfur and ensured good electronic conductivity. The electrochemical performance of the as‐prepared S/cPAN/carbon multi‐composite with 51% sulfur loading was tested in a high concentration LiTFSI electrolyte. Notably, the electrochemical performance of the comparatively higher sulfur containing S/cPAN/carbon multi‐composite was influenced by the common ion effect and viscosity of the electrolyte. This implies that the chemical pre‐treatment may be beneficial to have versatile SPAN in respect to the morphological structure, sulfur content and porosity. Although, it may involve with the multi‐step process, time, and cost.

#### 2.2.2 Physical Mixing Synthesis

The physical approach is considered simple and scalable compared to other methods, and therefore, most SPAN materials have been prepared following this method. In this approach, ball milling is applied to the mixture of PAN or AN and elemental sulfur (in the presence or absence of a solvent) in various ratios, followed by thermal treatment at high temperature under inert conditions.^[^ ^33^, ^45^, ^73^, ^77^, ^90^, ^91^ ^]^ The SPAN materials prepared by this method possess good physical and chemical properties. The polymeric matrix of the composite can hold both elemental sulfur and PSs during reaction, thereby leading to better electrochemical activity and stability. Many researchers use binders for protecting their cathode and decreasing the rate of dissolution of PSs.^[^ ^38^, ^59^ ^]^

Although thermal treatment is the key step in the preparation of the SPAN materials, ball milling is applied for better mixing, which could lead to complete chemical reaction upon heating. Kim et al.^[^ ^45^ ^]^ prepared SPAN by applying the ball‐milling process at 300 rpm for 12 h followed by thermal treatment at 450 °C for 6 h under N_2_ atmosphere. Evidently, most of the elemental sulfur was chemically bonded into SPAN (Figure 8d). The sulfur was covalently adopted as short chains of elemental sulfur (C—S structure) in the PAN matrix, while the long chains containing lithium PSs (Li_2_S*_n_*_= 2….8_)^[^ ^92^, ^93^ ^]^ were soluble in the electrolyte, thereby resulting in the shuttling effect leading to fatal capacity fading.^[^ ^94^ ^]^

Jin et al.^[^ ^58^ ^]^ prepared SPAN material by a similar synthetic strategy but including an additional step, by placing the sample in a stainless‐steel vessel and autoclaving at 350 °C for 10 h before thermal treatment. However, the as‐prepared SPAN was characterized by X‐ray photoelectron spectroscopy (XPS) and Raman spectroscopy along with ^13^C solid‐state NMR to analyze the sulfur structure. As observed in the S 2p XPS spectrum (Figure 8e), the two peaks located at 161.6 and 162.2 eV were due to the C—S single bond, whereas the peak at 163.2 eV was attributed to the C—S bond which connected short‐chain organosulfides to the carbon backbone, and the peak at 164.3 eV indicated the S—S bond of short‐chain organosulfides.^[^ ^33^, ^41^, ^95^ ^]^ In the Raman spectra, the peaks at 460 and 530 cm^−1^ were attributed to the S—S bonds, the peaks at 805 cm^−1^ represented C—S stretching, and the peak at 930 cm^−1^ indicated the stretching of a six‐membered ring containing S—S bonds.^[^ ^33^, ^44^ ^]^ Based on the structural analysis, it was confirmed that the sulfur diradical chains were chemically attached with the positively polarized carbon atoms, thereby forming S—C bonds on one side. Subsequently, another free side bonded with other sulfur chains forming S—S bonds located between two adjacent polypyridine rings. The resulting atomic ratio of C/S (2.93) suggests that the ideal sulfur chain should be —[S]_n = 3_— (Figure 4). The cycling stability of as‐prepared SPAN exhibited a good charging/discharging behavior and capacity retention of 98.4% at the 100th cycle with ≈100% CE. The lithiation process started at 1.68 V in the first discharge process with a specific capacity of 2207 mAh g^−1^. The potential then increased to ≈2.2 V for further cycles, and discharge specific capacities were 1729 and 1702 mAh g^−1^ at the 2nd and 100th cycles, respectively, which are higher than the theoretical value.

Liu et al.^[^ ^43^ ^]^ synthesized S@pPAN under various vapor pressures to observe the effect of vapor pressure on its structure and activity. They observed that the degree of graphitization and hydrogen content in SPAN can be controlled by vapor pressure, and therefore, a suitable vapor pressure (5 MPa) can accelerate the thermal reaction between PAN and sulfur, thereby developing a better conductive molecular structure for SPAN. The electrochemical performance was also dependent on the synthesis of SPAN composites. For example, the initial discharge specific capacity of 1821 mAh g^−1^ was calculated to be higher for the sulfur mass in the SPAN composite, which was prepared under 5‐MPa pressure (S@pPAN‐5), and the superiority was confirmed for the relatively stable specific capacities at various current densities.

**Figure 9:**
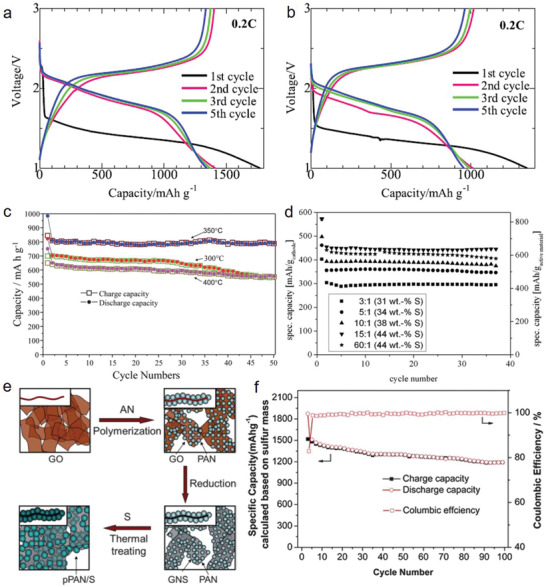
Voltage profiles of a) manually mixed SPAN, b) ball‐milled SPAN. Reproduced with permission.^[^ ^36^ ^]^ Copyright 2014, Elsevier. c) Cycle performance of SPAN prepared at 300, 350, and 400 °C. Reproduced with permission.^[^ ^96^ ^]^ Copyright 2016, Royal Society of Chemistry. d) Cycle stability of different SPAN materials (PAN: Sulfur = 1:3, 1:5, 1:10, 1:15, and 1:60) at 0.1 C. Reproduced with permission.^[^ ^98^ ^]^ Copyright 2012, Elsevier. e) Schematic of the in situ polymerization and synthesis of as‐prepared *_p_* PAN–S/GNS composite, and f) cycling performance of as‐prepared *_p_* PAN–S/GNS containing 4 wt% graphene at 0.1 C. Reproduced with permission.^[^ ^37^ ^]^ Copyright 2012, Elsevier. Insets: Cross‐sectional views of the corresponding samples.

Although several researchers have prepared SPAN materials by applying ball milling for better mixing, the ball‐mill treatment has been considered to be unfavorable owing to the electrochemical performance. Konarov et al.^[^ ^36^ ^]^ prepared two samples of SPAN by simple ”manual” mixing and by ball mill mixing for comparison. They observed better electrochemical performance and surface morphology in the manually mixed composite than in the ball‐milled composite. The relatively unchanged surface morphology with large‐sized particles in the ball‐milled sample suggests that the coagulation of sulfur occurred during thermal treatment. In contrast, reduced particle size (<100 nm) with uniform distribution was observed in the manually mixed composite. The comparison of discharge/charge profiles at 0.2 C revealed the superiority of the manually mixed sample (**Figure** **9**a) compared to the ball‐milled sample (Figure 9b). For example, the discharge capacity of the manually mixed SPAN was 1343 mAh g^−1^, which was considerably higher than the ball‐milled SPAN (950 mAh g^−1^). Additionally, the average discharge voltage (1.87 V) was higher than that of the ball‐milled SPAN (1.75 V).

Temperature is considered an important parameter not only for complete reaction but also for controlling the sulfur content in the prepared SPAN samples. Wang et al.^[^ ^96^ ^]^ prepared SPAN at various temperatures after ball milling for 6 h. The temperature‐dependent changes in chemical structure were measured by FT‐IR, and the results suggest that PAN and sulfur do not react between 120 and 150 °C. From 200 to 250 °C, PAN forms aromatic ring units by forming C = C and C = N bonds.^[^ ^97^ ^]^ At temperatures higher than 300 °C, the grafting of sulfur to the PAN‐derived backbones occurs, together with thioamide formation, following the equation C≡N + H_2_S → S—C=NH_2_.^[^ ^30^, ^44^ ^]^ The higher intensity of the FT‐IR spectrum between 350 and 400 °C compared to the spectrum at 300 °C indicates that the bond formation to the final structure may increase. Among all SPAN composites, the 350 °C‐treated sample exhibited the maximum initial discharge capacity of 984 mAh g^−1^, with a stable capacity (98.1%) after 50 cycles, which was relatively better than the other two samples prepared at 300 and 400 °C (Figure 9c). This indicates that the SPAN composite prepared at 350 °C was optimized to maintain balance between electrochemical activity and sulfur content. Another simple strategy can be applied to maintain balance between electrochemical activity and sulfur content. By simply changing of the initial ratio of PAN:sulfur addition and treated by a constant thermal condition, the electrochemical activity and stability can be simultaneously increased. However, following thermal treatment, the intrinsic sulfur loading can remain unchanged, although the initial ratio is increased up to 1:60. Fanous et al.^[^ ^98^ ^]^ demonstrated a maximum capacity of 1429 mAh g^−1^ with 85% sulfur utilization at 0.1 C, when the sulfur loading was up to 44 wt% for a PAN:sulfur ratio of 1:15. However, the initial ratio of 1:60 with the same sulfur loading (44 wt%) exhibited a lower capacity (1396 mAh $g_{\mathrm{sulfur}}^{-1}$), with only an 83% sulfur utilization (Figure 9d). This was likely due to all sulfur being mostly chemically bonded to the PAN‐derived backbone in the 1:15 sample and contributing to the higher lithium storage.^[^ ^44^, ^99^ ^]^ However, the presence of particulate (free elemental sulfur) and covalent addition of sulfur to the microcavities and backbone, in the 1:60 sample leads to poor capacity and sulfur utilization. Moreover, a comparatively better electrochemical performance was observed for the as‐prepared SPAN composite with a similar amount of sulfur content (42 wt%) by Wang et al.^[^ ^75^ ^]^

Combining chemical and physical approaches, Yin et al.^[^ ^37^ ^]^ prepared a SPAN composite material (pPAN–S/GNS) via an in situ chemical polymerization method and subsequent sulfur addition with ball milling, followed by thermal treatment over different periods for controlling the sulfur content (Figure 9e). By this method, they sequentially attached ≈100 nm‐sized regularly arrayed PAN particles on the surface of graphene nanosheets (GNS). The composite with 4 wt% GNS exhibited a reversible capacity of 1500 mAh g^−1^ in the first cycle, which was equivalent to a sulfur utilization of 90%. Additionally, the capacity retention was relatively stable at a 0.1 C rate, which was even comparable to the capacity of 800 mAh g^−1^ obtained at 6 C. Moreover, the composite showed a high CE (≈100%) (Figure 9f). The mildly reduced graphene oxide (rGO) nanosheets containing the SPAN composite (referring to the pPAN–S/mGO–S) exhibited a slightly lower initial reversible capacity of 1400 mAh g^−1^, which corresponded to an S utilization of 83%.^[^ ^100^ ^]^ However, the excess amount of GNS content (8 wt%) in the composite exhibited poor cyclic performance due to the dissociation of sulfur from the composite,^[^ ^101^ ^]^ thereby indicating that the ratio of used component was the key factor in improving the electrochemical performance of the as‐prepared SPAN composite.

#### 2.2.3 Other Methods: Electrospinning

Electrospinning is a well‐established and advantageous method for the preparation of freestanding and flexible SPAN‐based electrodes.^[^ ^5^, ^7^, ^31^, ^39^, ^44^, ^57^ ^]^ It is likely the simplest way for generating 1D ultrathin fibers that can be exceptionally long in length and uniform in diameter. Furthermore, it is a continuous and controlled process suitable for producing high volumes, with flexibility in selecting the components, morphology, structure, and functionality.

**Figure 10:**
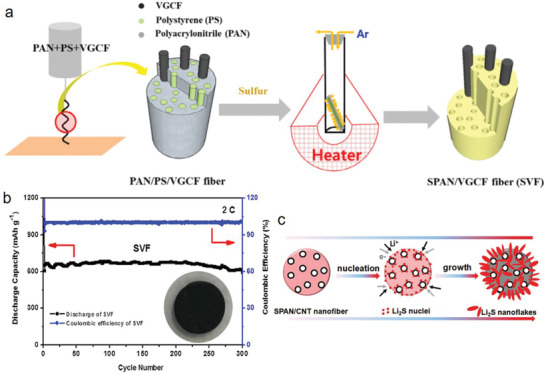
a) Schematic of the steps for fabricating SPAN composite. b) Cycling performance and CE of SVF cell at a rate of 2 C; inset: Optical image of SVF electrode. Reproduced with permission.^[^ ^101^ ^]^ Copyright 2014, American Chemical Society. c) Schematic for the nucleation‐growth/decomposition of Li_2_S nanoflakes from the cross‐sectional view of the SPAN/CNT‐12 nanofibers. Reproduced with permission.^[^ ^44^ ^]^ Copyright 2011, American Chemical Society.

**Figure 11:**
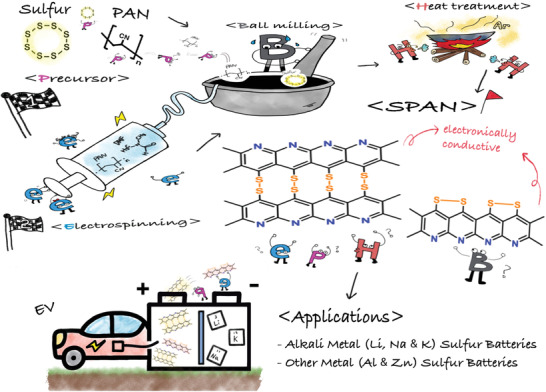
Synthetic pathways of SPAN electrodes and their applications in metal–sulfur batteries.

A freestanding porous SPAN fiber was prepared by applying a high voltage of 17 kV after the homogeneous mixing of vapor‐grown carbon fiber, polystyrene, and PAN, by high‐energy ball milling. This is followed by thermal treatment after soaking in a CS_2_/S (100:30, w/w) solution (**Figure** **10**a).^[^ ^102^ ^]^ The initial discharge capacity of the as‐prepared SPAN was 1814 mAh g^−1^ at 0.1 C, which was relatively higher than the theoretical value of sulfur. However, the capacity retention of the SPAN (SVF) cell was 903 mAh g^−1^ after 150 cycles at 1 C and 600 mAh g^−1^ with ≈100% CE after 300 cycles at 2 C (Figure 10b), although the as‐prepared SPAN was directly used as the electrode (Figure 10b inset). Moreover, the SPAN composite exhibited a superior rate capability than the as‐prepared SPAN particles, even at high C‐rates, which was due to the porous structure providing better accessibility between the electrode and electrolyte.^[^ ^42^ ^]^ However, evidently, the electrochemical process involved the nucleation and subsequent formation of Li_2_S nanoflakes (Figure 10c). At the initial discharge, Li_2_S possibly nucleated on the nanofiber surface. Subsequently, the Li_2_S nuclei turned into nanoflakes due to the continuous reduction of S. The decomposition of Li_2_S is completely reversible at the final charging cycle. To increase the battery performance, the hollow tubular SPAN nanofibers (denoted as H‐SPAN) were prepared at a comparatively low applied voltage of 15 kV (the distance between the coaxial needle and collector was 15 cm). The H‐SPAN electrode exhibited a discharge capacity of 1782.4 and 1250 mAh g^−1^ in the initial and subsequent cycles, respectively.^[^ ^103^ ^]^ Yun et al.^[^ ^18^ ^]^ have prepared an SPAN (CNF‐S) cathode via simple electrospinning of PAN followed by dipping in the slurry sulfur solution after carbonization at 1500 °C. The as prepared CNF physically adsorbed polysulfide (61.18 wt% sulfur) which reached a specific discharge capacity of 1139 mAh g^−1^ with the maintaining of 847 mAh g^−1^ after 100 cycles at a rate of 0.1 C. In addition, this cathode showed significantly lower polysulfide dissolution. Therefore, it implies that the electrospinning method is also effective to prepare SPAN. Although, it is difficult to understand that the sulfur may not be covalently bonded to the carbon matrix, PAN, while electrospinning does not affect the PAN upon high voltage application except formation of 1D nanofibers shape. We summarize and illustrate the typical synthetic routes of SPAN and its application as cathode material in MSBs for at a glance understanding (**Figure** **11**).

## 3 Sulfurized Polyacrylonitrile Composites

The preparation of composites together with conductive additives aiming to improve the electronic conductivity is one of the best ways to promote the electrochemical performance and stability of SPAN.^[^ ^34^, ^104^ ^]^ The aim of making a SPAN composite includes providing high surface area, morphological benefits, superior electronic conductivity, stability, and wide potential windows. Various materials such as nanocarbon (activated carbon, graphene, and carbon nanotubes (CNTs)), transition metals oxides, polymers, and sulfides have already been used for their preparation.

### 3.1 Carbon Nanotubes/Sulfurized Polyacrylonitrile Composite

**Figure 12:**
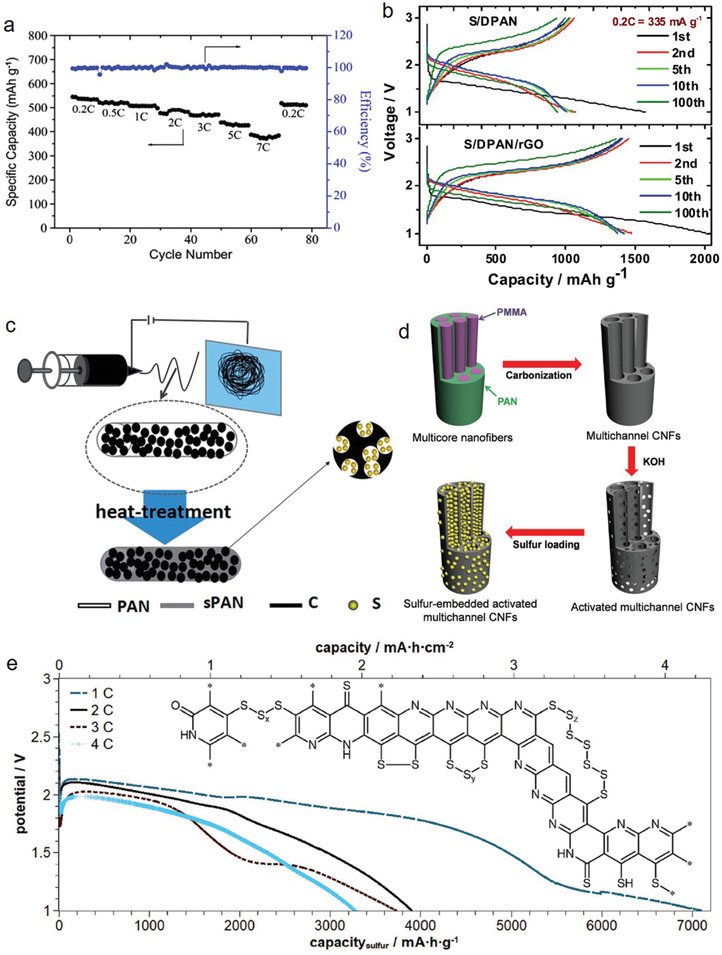
a) Cycling and efficiency response at various discharge rates of MWCNTs‐containing ternary composite. Reproduced with permission.^[^ ^107^ ^]^ Copyright 2014, Wiley‐VCH. b) Voltage profiles of S/PAN (upper) and S/DPAN/rGO (lower) composites. Reproduced with permission.^[^ ^40^ ^]^ Copyright 2017, Elsevier. c) Schematic of electrospinning preparation process. Reproduced with permission.^[^ ^120^ ^]^ Copyright 2014, Wiley‐VCH. d) Illustration of the sequential fabrication steps for the S‐a‐MCNF. Reproduced with permission.^[^ ^46^ ^]^ Copyright 2020, Royal Society of Chemistry. e) Structure of SPAN and initial discharge curves at 1, 2, 3, and 4 C in DME/DOL/DMTS (1:1:2) containing 1 m LiTFSI. Reproduced with permission.^[^ ^122^ ^]^ Copyright 2017, Elsevier.

CNTs have exceptional electrical, thermal, and mechanical properties that attract significant attention for using in composite materials.^[^ ^105^ ^]^ For example, introducing CNTs in sulfur electrode increases the overall electrochemical performance, including rate capability and cycle life.^[^ ^70^, ^106^ ^]^ Wang et al.^[^ ^44^ ^]^ prepared a 3D conductive nanofiber network of SPAN plus CNTs for Li–S cells. The CNTs and PAN composite was formed in a nanofibrous network through electrospinning, to provide fast charge‐transport and accommodation for volume expansion during the charge/discharge process. The electrochemical properties of the as‐prepared composite were controlled by changing the PAN and CNTs ratio. However, in the discharge–charge profiles, the SPAN/CNT‐12 (mass ratio of CNTs to PAN was 12 wt%) produced a high initial specific discharge capacity of 1884 mAh g^−1^ compared to that of pure SPAN (1767 mAh g^−1^), and the reaction kinetics were improved by the increased reversible capacity with a high CE (≈100%) in the subsequent cycles. The specific capacity of 1180 mAh g^−1^ was observed after 800 cycles with approximately 100% CE at the current density of 800 mA g^−1^. Strong bonding between the PAN backbone and short —[S]_*n* = 2,3_— chains enabled a highly reversible redox process. A ternary composite of multi‐walled CNTs (MWCNTs) and block PAN was prepared in the presence of sulfur, exhibiting exceptional sulfur utilization (≈95.3%) with a capacity retention of ≈96.5% at the 100th cycle.^[^ ^107^ ^]^ The charge/discharge efficiency of the ternary composite exhibited an initial capacity of 545.2 mAh g^−1^ at 0.2 C, which reduced to 386.7 mAh g^−1^ at 7 C, corresponding to 71% of the initial reversible capacity (**Figure** **12**a). Yin et al.^[^ ^70^ ^]^ introduced PAN onto MWCNTs via in situ polymerization, to develop a core‐shell structure, followed by the incorporation of sulfur via low‐temperature pyrolysis (pPAN–S@MWCNT). This composite, with 48% sulfur content, exhibited an initial discharge capacity of 697 mAh g^−1^ (1394 mAh g^−1^) with an 85% capacity retention after 50 cycles at 0.1 C.

### 3.2 Graphene/Sulfurized Polyacrylonitrile Composite

Graphene is a 2D and single‐atom‐thick sheet of carbon with a honeycomb lattice structure.^[^ ^108^ ^]^ Graphene has attracted significant attention as an ideal carbon material in various fields owing to its exceptional properties such as large surface to mass ratio, high thermal conductivity, mechanical strength, and electronic conduction.^[^ ^109^, ^110^, ^111^, ^112^ ^]^ The graphene matrix can provide effective ion conductivity, porous structure and increases structural stability of the SPAN composite system, resulting in excellent electrochemical properties of the SPAN cathode. Additionally, oxidized form of graphene (GO) might be able to minimize the polysulfides’ dissolution and their shuttle.^[^ ^94^ ^]^ Furthermore, the use of graphene in sulfur composite preparation could confirm the maximum utilization of sulfur moiety.^[^ ^113^ ^]^ A sulfur/dehydrogenated polyacrylonitrile (S/DPAN) composite was dispersed on rGO via self‐assembly in the presence of cetyltrimethylammonium bromide under simple sonication treatment, and the final product is denoted as S/DPAN/rGO. The results of morphological analysis revealed that the S/DPAN particles were sandwiched between the rGO layers, with a considerably high electric conductivity of 8 × 10^−4^ S cm^−1^ compared to the rGO‐free S/DPAN composite (≈10 × 10^−12^ S cm^−1^). The resulting composite exhibited a capacity of 1490 mAh g^−1^, which was 90% of its theoretical capacity and substantially higher than the S/DPAN (1000 mAh g^−1^) after the first cycle (Figure 12b). Li et al.^[^ ^114^ ^]^ prepared a SPAN composite (denoted as SPAN/RGO) with a 100 nm particle size, which was deposited on the surface of 3 wt% graphene. The 44 wt% S containing SPAN/RGO exhibited ≈85% retention of the initial reversible capacity of 1467 mAh g^−1^ at approximately 100 cycles and 1100 mAh g^−1^ after 200 cycles at a constant current rate of 0.1 C. However, the pure SPAN composite exhibited rapid capacity decay following 40 cycles, thereby reaching no capacity at the 100th cycle with the same current rate. Alternatively, graphene was introduced to SPAN composite to improve its electrochemical performance and stability. A sample prepared with GNS‐wrapped hierarchical pPAN–S microspherical particles (pPAN–S@GNS) exhibited a high reversible capacity of 1449.3 mAh g^−1^ during the second cycle, retaining 88.8% of the initial discharge capacity with ≈100% CE.^[^ ^115^ ^]^ The S/PAN/graphene composite (sulfur content 47.3%) was prepared via ball‐milling method followed by thermal treatment at a low temperature. This SPAN exhibited an improved rate capability of 360 mAh g^−1^ (761 mAh g^−1^) at 4.0 C, with a 77% capacity retention over 100 cycles at 0.1 C in a Li–S system.^[^ ^116^ ^]^

### 3.3 Carbon/Sulfurized Polyacrylonitrile Composite

To improve the electrochemical performance and cycle stability of sulfur cathode, SPAN has been combined with carbon as it is a conductive, mesoporous/microporous, and inexpensive material. Carbon can provide better electrochemical performance when composited with the SPAN.^[^ ^10^, ^117^, ^118^, ^119^ ^]^ For improved cycling stability, the S/C/PAN nanofiber was prepared as a long‐life and high‐capacity cathode material for a Li–S battery system (Figure 12c). The S/C/PAN composite cathode exhibited a high reversible capacity of 1179 mAh g^−1^ at a current rate of 200 mA g^−1^ after a few cycles and a good rate capability with 616 mAh g^−1^ at 4.0 A g^−1^. In addition, it had a high CE of ≈100%, with 60% capacity retention over 400 cycles in a carbonate‐based electrolyte.^[^ ^120^ ^]^ In the composite nanofibers, the sulfurized PAN matrix operated as ionic channels to allow the Li^+^ to react with the S/C nanoparticles and as a shield to prevent the S/C from contacting the electrolyte, thereby leading to higher stability.

As a current collector, ball‐milled carbon fibers (CFs) were grafted onto SPAN following fabrication using the electrostatic flocking method. SPAN electrodes with the CFs current collectors demonstrated better electrochemical performance than those with aluminum current collectors. The SPAN electrodes with CF current collectors yielded a discharge capacity of 1658 mAh g^−1^ at a loading weight of 0.5 g, which was higher than the electrode with an Al current collector (1536 mAh g^−1^).^[^ ^121^ ^]^

### 3.4 Polymer/Sulfurized Polyacrylonitrile Composite

Various conduction polymers have been used for preparing SPAN materials.^[^ ^34^, ^46^, ^59^, ^80^, ^122^ ^]^ Along with PAN, polymethyl methacrylate (PMMA) is extensively used for synthesizing electroactive composites.^[^ ^34^, ^46^, ^80^, ^122^ ^]^ Pure PAN is crystalline,^[^ ^123^ ^]^ and therefore, it hinders the incorporation of sulfur into the compact PAN (in the form of fibers), leading to lower sulfur contents (approximately 10 wt%). Therefore, blending with PMMA can reduce the crystallinity and increase the porosity of PAN, leading to the incorporation of sulfur and subsequent improved electrochemical performance. To obtain an enhanced electrochemical performance in the Li–S system, Lee et al.^[^ ^46^ ^]^ prepared a SPAN material (named S‐a‐MCNF) through a facile single‐nozzle co‐electrospinning technique (Figure 12d). The S‐a‐MCNF exhibited exceptional performance (initial capacity of 1351 mAh g^−1^ and 847 mAh g^−1^ at a 0.2 C and 5 C rate, respectively) with the typical two plateaus in the discharge curves that were associated with the formation of long‐chain PSs (Li_2_S*_n_*, n = 4…8) at 2.3 V and short‐chain PSs (Li_2_S_2_ and Li_2_S) at 2.1 V. Moreover, a 920 mAh g^−1^ capacity was maintained after 300 cycles, with a CE higher than 99.5%. The PMMA/PAN‐based fibrous SPAN (42 wt% of sulfur), modified with dimethyl trisulfide cells (Li/DMTS/SPAN), exhibited a high initial capacity of 7100 mAh g^−1^ (Figure 12e), together with a high rate capability up to 8 C and efficient cycle stability.

### 3.5 Wrapping/Coating by Single Molecule/Sulfurized Polyacrylonitrile Composite

**Figure 13:**
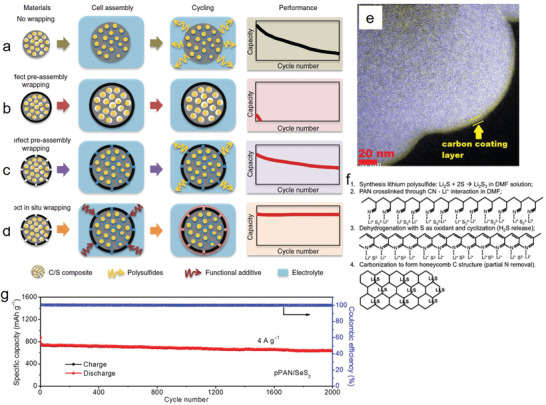
Schematic of the in situ wrapping strategy for CMK‐3/S@PANS@TPS. a) No‐wrapping case, exhibiting severe capacity decay during cycling, b) perfect‐wrapping case, exhibiting poor overall performance due to the lack of electrolyte in the active material, and c) imperfect‐wrapping case, exhibiting improved cycle stability compared with the no‐wrapping case. d) Perfect post‐assembly in situ wrapping of the cathode material, exhibiting ideal cycle stability using a blocking PSs shuttle while allowing for electrolyte infiltration in the active material. Reproduced with permission.^[^ ^124^ ^]^ Copyright 2015, American Chemical Society. e) Elemental mapping of C and S showing carbon‐coating onto the C@S/PAN composite. Reproduced with permission.^[^ ^125^ ^]^ Copyright 2019, American Chemical Society. f) Proposed synthesis route for creating Li_2_S–carbon cathode materials. Reproduced with permission.^[^ ^132^ ^]^ Copyright 2020, Elsevier. g) Prolonged cycle life at 4 A g^−1^ of pPAN/SeS_2_. Reproduced with permission.^[^ ^136^ ^]^ Copyright 2016, Royal Society of Chemistry.

A different approach to improve the electrochemical performance is the manufacturing of a SPAN composite covered by an outer layer material, such as, single molecules or polymers, through a wrapping or coating technique. This type of nanoarchitecture involves coating SPAN with an outer layer of porous material. The porous layer allows the easy access of reactant ions or electrons to trigger active desired electrochemical reactions, while increasing the durability of the SPAN composite.^[^ ^124^, ^125^, ^126^ ^]^ To increase the performance of the SPAN composite cathode, Hu et al.^[^ ^124^ ^]^ developed an in situ wrapping technique that blocked the polysulfide diffusion into the electrolyte. A mesoporous CMK‐3/sulfur composite was initially prepared as the core of this architecture in this technique. Subsequently, a pan layer with ≈40 nm thickness was grown on CMK‐3/sulfur via in situ free radical polymerization reaction,^[^ ^124^ ^]^ followed by the sulfurization of the PAN layer under thermal treatment^[^ ^29^, ^70^ ^]^ to obtain CMK‐3/S@PANS, and placed into coin cells as the cathode. The TPS layer (≈15 nm) was then expected to form on the PANS layer (denoted by CMK‐3/S@PANS@TPS) by the sulfurization of triphenylphosphine, which was placed in the electrolyte earlier. The structure of various CMK‐3/S particles in different wrapping conditions were illustrated, and the corresponding performance is shown in **Figure** **13**a–d. The in situ‐wrapped CMK‐3/S@PANS@TPS exhibited an initial specific discharge capacity of 1246 mAh g^−1^ at the rate of 0.25 C. Importantly, the capacity decay rate was 0.034% per cycle, and the CE was maintained at 96.2 and 98.4% at the 1 and 2 C rates, respectively, after 1000 cycles. Moreover, Peng et al.^[^ ^125^ ^]^ prepared a carbon‐coated SPAN composite (denoted as C@S/PAN) via a novel solution processing method. A single molecule, sucrose, was used in the fabrication of a carbon shell with ≈2 nm thickness onto a SPAN composite (Figure 13e). The prepared composite exhibited a high initial discharge capacity of 1416 mAh g^−1^ with a clear plateau in the range of 1.0–1.6 V, which remained at ≈89% after 200 cycles at a current rate of 0.1 C.

### 3.6 Metal Oxides or Sulfide/Sulfurized Polyacrylonitrile Composites

The addition of metal oxides or metal sulfides can effectively enhance the electrochemical performance of SPAN composite materials.^[^ ^127^, ^128^ ^]^ As additives, metal oxides can provide better conductivity, together with the advantages such as fast electrochemical effects. Metal oxides have also exhibited the adsorbing ability of PSs in secondary battery systems.^[^ ^129^ ^]^ To improve the electrochemical performance and the physical properties, an Mg_0.6_Ni_0.4_O‐containing SPAN composite (referred to as S/PAN/Mg_0.6_Ni_0.4_O) has been prepared via wet‐ball milling.^[^ ^130^ ^]^ The as‐prepared composite S/PAN/Mg_0.6_Ni_0.4_O exhibited approximately two times higher specific surface area than the pure S/PAN. As expected, the S/PAN/Mg_0.6_Ni_0.4_O composite displayed improved electrochemical performance than the pure S/PAN.

The aim of introducing metal sulfides into SPAN is to mitigate the dissolution of PSs and shuttling during electrochemical cycling, thereby leading to superior electrochemical performance and stability.^[^ ^41^, ^131^, ^132^, ^133^ ^]^ Guo et al.^[^ ^134^ ^]^ have shown a new method for preparing a uniformly dispersed composite based on lithium sulfide in a carbon host‐like PAN. The interaction between the Li‐ions and uniformly distributed nitrile groups of the PAN backbone in DMF solution was utilized to control the distribution of Li_2_S in the host material (Figure 13f). Similarly, SeS*_x_* molecules were confined by nitrile groups in the carbonized PAN to mitigate the dissolution of PSs and polyselenide intermediates. The prepared SeS_0.7_/CPAN composite maintained a reversible capacity of 780 mAh g^−1^ for 1200 cycles at a current density of 600 mA g^−1^.^[^ ^135^ ^]^ Moreover, it exhibited a high rate capability as its capacity was retained at ≈50%, when current density was increased by 100 times (60 mA g^−1^ to 6 A g^−1^). Furthermore, the pPAN/SeS_2_ prepared by applying a high voltage (17 kV) exhibited a discharge capacity of 871 mAh g^−1^ at 4 A g^−1^, which remained at 633 mAh g^−1^ after 2000 cycles maintaining a very low capacity decay rate (0.014% per cycle) (Figure 13g).^[^ ^136^ ^]^

Silicon (Si) or silicon (SiO_2_) is a popular electrode material for LIBs due to its high theoretical capacity of 3579 mAh g^−1[^ ^137^, ^138^, ^139^, ^140^ ^]^ or 4200 mAh g^−1^.^[^ ^141^ ^]^ Further, safety performance and suitable operating potential make it promising candidate for practical batter application.^[^ ^142^, ^143^ ^]^ However, the formation and decomposition of the Li_15_Si_4_ phase during charge and discharge process is accompanied with a large volume change (≈300%) of Si which leads to a low CE and rapid capacity fading.^[^ ^144^, ^145^, ^146^ ^]^ To overcome this problem, recently, the fabrication of composite materials containing Si nanoparticles in carbon host matrices is considered as a good strategy. Further, the addition of functional polymer like SPAN has also been considered as alternative strategy and draw considerable attention as practically applicable pathway. For example, Cengiz et al.^[^ ^147^ ^]^ have prepared a sPAN‐SiO*_x_* via electrospinning method which soaked into 0.03 m solution of Li_2_S_4_ in DOL. The Li_2_S_4_ adsorbed sPAN‐SiO*_x_* showed charge–discharge profile consisting of two typical plateau behavior of Li–S battery at discharge curve which corresponding to the transition of sulfur to long‐chain polysulfides and long‐chain polysulfides to short‐chain polysulfides, respectively. The sPAN‐SiO*_x_* containing cell gained more electrons compared to others, while longer plateau is the indication of more active material utilization. As expected, it showed first discharge capacity of 1321 and 1100 mAh g^−1^ for the cells prepared with sPAN‐SiO*_x_* and sPAN, respectively. Therefore, better performance resulting from the polysulfide adsorption effect of SiO*_x_*, N‐rich structure and the synergistic effect of sPAN‐SiO*_x_* along with flexible expansion of SiO*_x_*.

### 3.7 Heteroatom‐Doping in Sulfurized Polyacrylonitrile

Heteroatom‐doping in carbon materials is one of the best methods to improve the electrochemical performance^[^ ^148^, ^149^, ^150^, ^151^, ^152^, ^153^, ^154^, ^155^, ^156^ ^]^ because of several advantages such as, wettability, superior conductivity, charge transport, capacitive performance, and energy/power densities.^[^ ^157^, ^158^, ^159^, ^160^, ^161^ ^]^ Further, the doped heteroatoms can effectively modulate the electronic and chemical properties by acting superior active sites, increasing defects and enhancing charge‐transfer ability.^[^ ^162^, ^163^, ^164^ ^]^ More reports have been published on metallic doping for SPAN materials than on nonmetallic doping. Ma et al.^[^ ^74^ ^]^ investigated the electrochemical performance and stability of iodine‐doped SPAN (referred to as I‐S@pPAN) prepared by a ball‐milling process in the presence of iodine followed by thermal treatment at 300 °C, for both sodium–sulfur (Na–S) and potassium–sulfur (K–S) batteries at room temperature. The initial capacity of I‐S@pPAN containing ≈42% of sulfur was 1787 mAh g^−1^, which was higher than that of S@pPAN (1447 mAh g^−1^). The capacity was maintained at 850 mAh g^−1^ at a rate of 0.1 C and 674 mAh g^−1^ at a rate of 0.5 C for the I‐S@pPAN electrode after 100 and 500 cycles, respectively, with a CE above 99%. According to their results, the beneficial acceleration of Na^+^ intercalation and superior conductivity (5.90 × 10^−10^ S cm^−1^) resulted from the I‐doping of the I‐S@pPAN.^[^ ^165^ ^]^

It is necessary to design tellurium‐ or selenium‐doped SPAN materials to further enhance the electrochemical reaction kinetics.^[^ ^48^, ^154^, ^155^ ^]^ Being in the same group as sulfur, they could not only deliver better rate performance in the doped composite materials but also could act as active materials for contributing capacity through their own redox reactions at a molecular‐level distribution through Te—S/Se—S bonds.^[^ ^166^, ^167^ ^]^ Li et al.^[^ ^48^ ^]^ used Te as a eutectic accelerator to prevent the dissolution of PSs under a “dissolution–deposition” mechanism and accelerate the redox conversion in their Te_0.04_S_0.96_@pPAN composite. The —[S]*_n_*_≤ 4_— chains containing the as‐prepared composite exhibited high capacities of 1507 and 861 mAh g^−1^ at 0.1 and 10 A g^−1^, respectively, in ether electrolyte with a 0.05% capacity decay per cycle. Additionally, the Te_0.04_S_0.96_@pPAN exhibited more than twice the Li^+^ diffusion coefficient compared to that of the S@pPAN in a Li−S battery. This composite also delivered the capacities of 1111 and 601 mAh g^−1^ at 0.1 and 6 A g^−1^, respectively, in ether electrolyte in a Na−S battery.^[^ ^154^ ^]^ Similarly, Se was also used as a eutectic accelerator in a Se_0.05_S_0.95_@pPAN composite.^[^ ^155^ ^]^ The as‐prepared composite demonstrated a higher initial capacity of 1345 mAh g^−1^, which corresponded to an active material utilization of 86.2%, although S@pPAN exhibited only 1136 mAh g^−1^ (equivalent to 67.8% active material utilization). After 150 cycles, Se_0.05_S_0.95_@pPAN and S@pPAN maintained a capacity of 654 and 296 mAh g^−1^ with 81% and 50% capacity retention, respectively, based on the capacity in the second discharge.

Based on the above discussion, the SPAN composites have shown better charge/discharge profile, stability, and CE than the pristine SPAN. Although, I‐doped SPAN showed better electrochemical performance, the field of heteroatom‐doping may not be tremendously studied yet. For example, boron and oxygen doping still remain out of focus. Additionally, halogen group atoms can also be taken into the consideration.

## 4 Electrochemical Performance of Sulfurized Polyacrylonitrile as a Cathode

### 4.1 Lithium Systems

**Figure 14:**
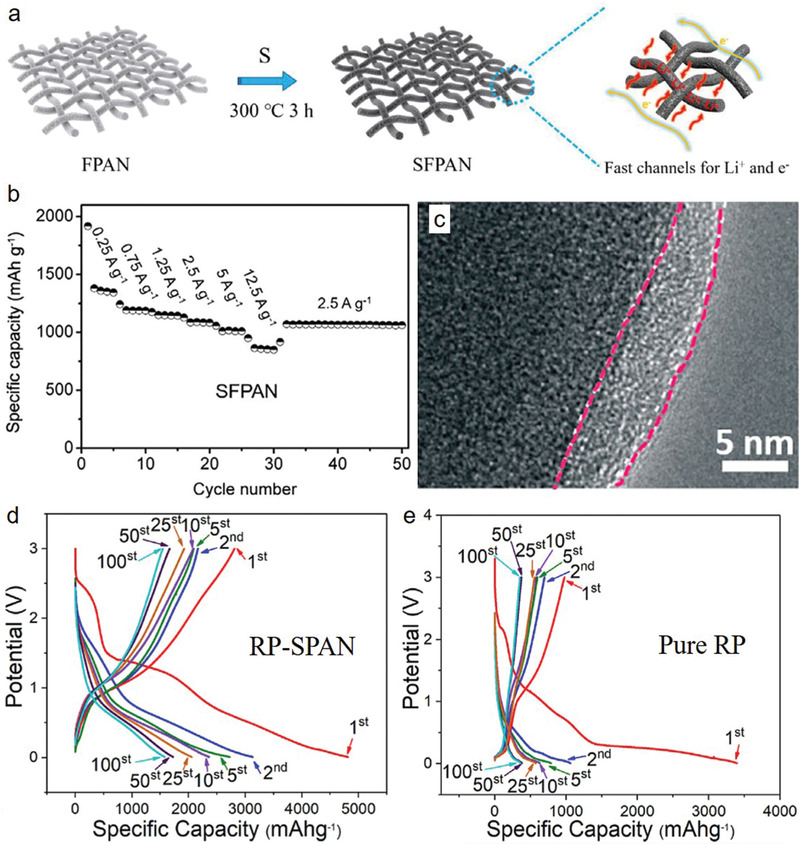
a) Graphic illustration of the formation process of the SFPAN composite. b) Cyclic capacities of SFPAN at different rates. Reproduced with permission.^[^ ^171^ ^]^ Copyright 2016, Elsevier. c) TEM image of the S‐PAN after 100 cycles in the 1 wt%‐LiBOB electrolyte. Reproduced with permission.^[^ ^173^ ^]^ Copyright 2019, Elsevier. Typical charge/discharge profiles at 0.1 A g^−1^ for the d) RP‐SPAN hybrid, and e) pure RP. Reproduced with permission.^[^ ^174^ ^]^ Copyright 2016, Springer Nature.

SPAN composites are also promising cathode materials for MSBs because of high sulfur utilization, less dissolution of PSs, and their conductive nature. The improved physical properties and electrochemical performance are due to sulfur immobilization by covalent chemical anchoring.^[^ ^167^ ^]^ In addition, it can be used with various types of electrolytes, such as, solid,^[^ ^155^ ^]^ polymer,^[^ ^169^ ^]^ or gel electrolytes.^[^ ^170^ ^]^ A novel 1D SFPAN composite can provide fast migration of ions and electrons, facilitating the electrochemical performance at high rates for Li–S batteries (**Figure** **14**a). FT‐IR and XPS analyses revealed that sulfur was covalently bonded on the carbon backbone of PAN in the SFPAN composite, enabling the high reversible specific capacity of ≈1200 mAh g^−1^ at 0.3 A g^−1^ after 400 cycles with a high rate capability up to 12.5 A g^−1^ (≈850 mAh g^−1^) (Figure 14b).^[^ ^171^ ^]^ S@PAN‐DG was prepared with the support of a vulcanization accelerator agent (diphenyl guanidine), which improved the sulfur content of 14 wt% together with the conductivity and specific surface area compared to that of pure SPAN.^[^ ^172^ ^]^ Therefore, the capacity retention was 90.1% at a rate of 1 C after 200 cycles for S@PAN‐DG, indicating good electrochemical performance at high areal loading. Jin et al.^[^ ^173^ ^]^ improved the cycling performance of an S‐PAN cathode in a Li–S battery via an in situ wrapping method using lithium bis(oxalato)borate in electrolyte. TEM and XPS analyses revealed that a wrapping layer with a thickness of ≈5 nm was formed after 100 cycles on the S‐PAN (Figure 14c), thereby leading to the improvement of the capacity retention ratio from 38% to 62%. Baboukani et al.^[^ ^174^ ^]^ prepared a red phosphorus‐SPAN (RP‐SPAN) hybrid anode material for LIBs through a ball‐milling and electrostatic spray deposition techniques. The binder‐free RP‐SPAN anode exhibited a highly reversible specific capacity of 1605 mAh g^−1^ at 100 mA g^−1^. Moreover, the RP‐SPAN displayed specific discharge/charge capacities of 4810/2809 and 1605/1579 mAh g^−1^ (Figure 14d) which were considerably higher than that of pure RP (3393/976 and 358/352 mAh g^−1^) (Figure 14e) at the 1st and 100th cycle, respectively, at 0.1 C. The S/CPAN‐800 composite^[^ ^175^ ^]^ was prepared using an SBA‐15 template producing a high specific surface area and a highly ordered mesoporous structure with moderate nitrogen content, thereby exhibiting a comparatively better enhanced capacity retention of 862 mAh g^−1^ at 0.1 C after 100 cycles. Similar results were observed for *p*PAN‐KB/S, which contained a high amount of sulfur (≈72%) due to high surface area (727 m^2^ g^−1^). This compound delivered a capacity of 866 mAh g^−1^ after 100 cycles at 0.5 C.^[^ ^176^ ^]^ Recently, the in situ growth of ZIF‐67 on PAN‐CNTs films with CoS_2_ (denoted as CoS_2_–SPAN–CNT) was used in Li–S batteries.^[^ ^177^ ^]^ This material exhibited a reversible capacity of 880 mAh g^−1^ with approximately 100% CE after 400 cycles at a rate of 1 C. The S/rSP@SPAN cathode with high sulfur loading (54.5%) demonstrated a high rate capability of 492 mAh g^−1^ at a rate of 10 C.^[^ ^177^ ^]^

### 4.2 Sodium Systems

Owing to the similarities in electrochemical behavior and mechanism between Li–S and Na–S and reduced cost, room temperature Na–S batteries have received great interest, since 2006.^[^ ^179^, ^180^ ^]^ In a Na‐battery system, SPAN materials can be used in pure, doped, and composite forms. It showed exceptional electrochemical activity, with first discharge capacity of 1473 mAh g^−1^ at 0.01 C by pristine SPAN web^[^ ^181^ ^]^ and high rate capability (maintaining 327.5 mAh g^−1^ after 500 cycles at 5 A g^−1^) and CE by a composite FeS/SPAN‐HNF.^[^ ^133^ ^]^ Wang et al.^[^ ^152^ ^]^ developed a pure SPAN composite in a room temperature Na–S battery system for the first time. Their materials exhibited ≈100% charge/discharge efficiency and maintained ≈500 mAh g^−1^ up to the 18th cycle. Recently, Huang et al.^[^ ^103^ ^]^ reported on H‐SPAN in a room temperature Na–S battery system with improved cycling performance. After 200 cycles, a high reversible capacity of 717 mAh g^−1^ at a rate of 0.1 C was attained.

A Se‐doped SPAN (denoted as Se_0.08_S_0.92_@pPAN) was used for rechargeable Na batteries by Wang et al.^[^ ^153^ ^]^ Unique properties such as high electronic conductivity and high selenium density are expected to improve the active material utilization.^[^ ^182^ ^]^ Therefore, selenium doping in Se_0.08_S_0.92_@pPAN was designed to exploit the high capacity and conductivity originating from sulfur and selenium synergy, thereby resulting in a specific capacity of 770 mAh g^−1^ at 0.4 A g^−1^ for over 500 cycles and exceptional CE (≈100%).

Another dopant element, iodine, was introduced in the PAN organic framework for application in Na–S batteries.^[^ ^74^ ^]^ The iodine doping onto PAN increased the electrical conductivity and Na^+^ diffusion coefficient by approximately two orders compared to the pristine SPAN, exhibiting a high capacity of 994 mAh g^−1^ at a high rate, that is, 2 C, as well as, a stable cycling performance with low capacity decay rate (0.029% per cycle) for over 500 cycles. In the proposed reaction mechanism, the broken —[S]_2≤_ *_n_*_≤4_— chains, double‐bond carbon species (C = N or C = C), and doped iodine react with Na^+^ offering extra capacity for the irreversible first discharge. Most of the discharged product is converted to initial functional groups in the first charge process. However, a small portion of some functional groups may not remove the Na^+^ (C—N—Na or C—C—Na), resulting in a capacity loss during the first charge process. The discharged and charged products of the first cycle proceed with reversible conversion reactions in the subsequent cycles.

Haridas et al.^[^ ^133^ ^]^ recently developed a FeS/SPAN‐HNF fiber matrix by the sulfurization of Fe_2_O_3_ and PAN fibers through electrospinning at a voltage of 20 kV followed by a thermal treatment. The authors showed that the comparatively better conductive FeS/SPAN‐HNF performed as a dual‐functional matrix that was able to control the volume expansion and sulfur dissolution of FeS and SPAN. Consequently, it exhibited a high reversible capacity of 782.8 mAh g^−1^ at 200 mA g^−1^, with an exceptional rate capability (maintaining 327.5 mAh g^−1^ at 5 A g^−1^, up to 500 cycles). During post‐cycling analysis, the high‐resolution TEM image of FeS/SPAN‐HNF and XRD confirmed the formation of NaFeS_2_ following sodiation reaction.

**Figure 15:**
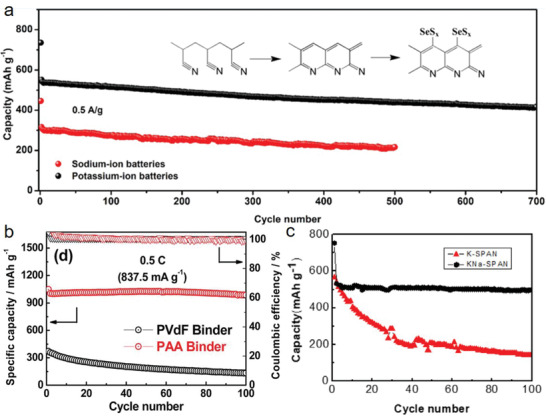
a) Long‐term cycling performance of the OC/SeS_2_ electrode at 0.5 A g^−1^ over 700 cycles for Na‐ion batteries and over 500 cycles for K‐ion batteries. Reproduced with permission.^[^ ^183^ ^]^ Copyright 2007, Elsevier. b) Cycle life test of the SPAN electrode with PVDF and PAA binders. Reproduced with permission.^[^ ^35^ ^]^ Copyright 2018, Royal Society of Chemistry. c) Discharge capacity of a KNa–SPAN battery and K–SPAN battery. Reproduced with permission.^[^ ^189^ ^]^ Copyright 2011, American Chemical Society.

Similarly, an organic carbon/selenium sulfide (OC/SeS_2_) composite was used as a cathode for a Na–S battery system.^[^ ^183^ ^]^ The high electronic conductivity and miscibility of Se and S and high electrical conductivity and chemical binding with SeS_2_ of the N‐doped carbon network were expected to improve the active material utilization, thereby resulting in the good reversible capacity of 416 mAh g^−1^ after 700 cycles at a rate of 0.5 A g^−1^ (**Figure** **15**a).

### 4.3 Potassium System

As potassium has a lower reduction potential (−2.93 V) than sodium (−2.71 V), potassium‐based batteries can exploit a higher working potential. Furthermore, potassium is cheaper than sodium as it is abundantly available in the soil and oceans. Therefore, potassium–sulfur (K–S) batteries have emerged as a prospective future battery system, attracting considerable research interest. Chen et al. introduced the concept of a room temperature K–S battery for the first time in 2014.^[^ ^184^ ^]^ Although the first K–S concept experienced poor cycling stability, significant progress has been recently made because of the advancements in cathode engineering and studying of the K–S mechanism.^[^ ^185^, ^186^ ^]^ SPAN and its composite are also interesting materials for room temperature K–S batteries.^[^ ^74^, ^187^ ^]^ Iodine‐doped SPAN (I‐S@pPAN) exhibited a reversible capacity of 947 mAh g^−1^ at a 0.1 C rate in the room temperature K–S battery.^[^ ^74^ ^]^ Confined and covalent sulfur (CCS) was prepared by annealing PAN and sulfur at 450 °C, achieving a high surface area and conductivity.^[^ ^187^ ^]^ The 39.25% sulfur‐containing CCS exhibited a capacity retention of 86.3% over 300 cycles with a CE ≈100% at a voltage cut‐off of 0.8–3.0 V.

Hwang et al.^[^ ^35^ ^]^ reported that the activity of a cathode can be significantly improved by a simple change in the binder. By integrating SPAN and polyacrylic acid (PAA) binder, the cathode exhibited a high reversible capacity of 1050 mAh g^−1^, with exceptional cycling stability (95% retention after 100 cycles) at the rate of 0.5 C, a value significantly higher than that of the electrode using poly(vinylidene fluoride) (PVDF; initial discharge capacity of 370 mAh g^−1^ with only 22% after 100 cycles) (Figure 15b). However, research on K–S batteries is in its infancy stage, and extensive research efforts are necessary to optimize the performance of K–S and the understanding of its reaction mechanisms.

### 4.4 Other Systems

Mg–S battery systems might be less expensive, safer, and environment‐friendly due to the characteristics of Mg such as, natural abundance, low price, and higher expected safety than other metals. However, Mg‐based batteries are not comparable in terms of energy density with Li‐based batteries. Yang et al.^[^ ^188^ ^]^ showed the possibility of preparing conductive sulfur‐containing SPAN (denoted as CMS) cathode material that exhibited initial discharge capacity of 120 mAh g^−1^ against Mg anode. Following this attempt, there have not been any significant studies this far.

The Al–S battery is also a system that has attracted less attention because of its solubility issues and high irreversibility of AlS*_x_* phases that causes limited stability when the battery is cycled. To overcome these problems, Wang et al.^[^ ^78^ ^]^ reported on an easily prepared SPAN, that exhibited good cycling performance, using an electrolyte prepared by mixing 1‐ethyl‐3‐methylimidazolium chloride and aluminum trichloride (1:1.5) at 50 °C. The results reported on a second discharge capacity higher than the first discharge capacity (605 vs 320 mAh g^−1^) and superior rate performance that was achieved because of S—S bond breaking as well as both S and N atoms acting as the active sites for Al^3+^.

Zhang et al.^[^ ^189^ ^]^ prepared a SPAN cathode material for a K–Na/SPAN battery system. They used a liquid K–Na alloy as the anode to avoid dendrite growth on the anode during the charge process.^[^ ^190^ ^]^ Correspondingly, the electrochemical performance of the K–Na/SPAN battery system exhibited a high capacity of 513 mAh g^−1^, which was maintained at 490 mAh g^−1^ after 100 cycles, along with an exceptional CE (≈100%) at 35 mA g^−1^, whereas the K–SPAN battery exhibited only 140 mAh g^−1^ after 100 cycles (Figure 15c).

Si–S battery is rarely investigated battery system while it cannot match with sulfur cathode. Although Si anode exhibits high capacity and low electrochemical potential. However, Zhang et al.^[^ ^191^ ^]^ have developed a simple strategy to prepare Li–Si alloy anode by two step process. First, the made free‐standing Si/C film by electrospinning which lithiated in the second step by placing it in between Li‐foil and separator and subsequent adding of electrolytes during cell assembly. Then the lithiated Si/C film has used as anode and 43% sulfur contenting S@pPAN has used as cathode. The as assembled lithiated Si–S cell showed the initial discharge and charge capacities of ≈1750 and 1250 mAh g^−1^ at 1 C. In addition, it possesses a capacity retention of 88% and CE of ≈100% for the second cycle after 800 cycles which was much better than that of Li‐S cell.

It is observed that the specific capacity and the initial CE in Na–S and K–S systems (including other systems) are lower than that of Li–S system in SPAN cathode. For instance, Al–S showed much lower specific capacity and unstable initial CE than the Li–S.^[^ ^78^ ^]^ This is probably due to the much bigger ionic radius of Al^3+^ which requires the longer activation process. Further, at the initial few cycles, the bigger than Li ions like Na^+^ need to go through a barrier, which is probably related to desolvation or solvation shell distortion^[^ ^187^ ^]^ to accommodate the extremely small pore size to diffuse inside the micropores. Therefore, further study on molecular structure of SPAN is required to fully understand the overall performance of SPAN cathode including electrochemical mechanism. In addition, for increasing the practical capacity to the theoretical capacity, it is necessary to increase the sulfur contents in carbon backbone covalently. In this regard, new types of carbon backbone should be introduced which is similar to the PAN structure. For example, PANI could be a probable candidate. Tang et al.^[^ ^192^ ^]^ prepared a PANI derived ACA‐500‐S@PANi cathode which content 61% of sulfur and delivered a highly reversible capacity from the first cycle without over‐capacity in the first cycle. The **Table** **1** summarizes various SPAN materials and their battery performances for better visualization.

## 5 Electrolyte Compatibility with Sulfurized Polyacrylonitrile Cathode

When examining the history of LIBs, electrolytes are key components in determining battery performances. In MSBs, similar to LIBs, investigating electrolyte compatibility with SPAN cathode is necessary for improving the battery performances. Unquestionably, an optimized electrolyte system aids efficient and long‐term operation of a battery. To date, liquid‐based electrolytes are the extensively studied and applied electrolytes in current battery technologies. As summarized in previous sections, numerous publications have reported MSB systems with a variety of electrode materials; however, no systematic study has been conducted on the electrolyte and its role in the performance for SPAN cathode.

**Scheme 1:**
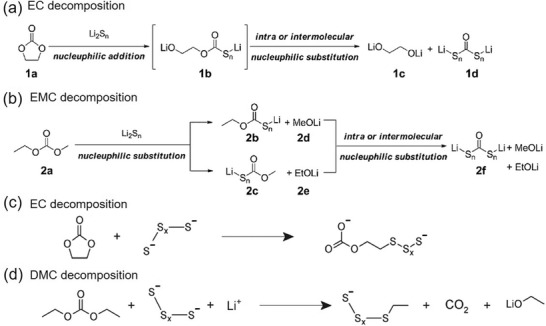
Possible reaction mechanism for the decpmposition of carbonate solvents by polysulfides. a,b) Reproduced with permission.^193^ Copyright 2016, Springer Nature. c,d) Reproduced with permission.^[^ ^194^ ^]^ Copyright 2013, Springer Nature.

The fundamental features of liquid electrolytes intrinsically depend on the nature of the alkali–metal salt dissolved and a polar aprotic organic solvent. There are two common categories of aprotic liquid electrolytes in battery applications: One is carbonate (ester)‐based and the other is ether‐based electrolytes. Carbonate‐based electrolytes are widely used in commercial LIBs due to their electrochemical stability with a wide range of working voltages and chemical stability with various electrode materials. However, the carbonate‐based electrolytes are not compatible (desirable) with a conventional Li–S battery (or MSBs) based on the conversion reaction of “S_8_ +2Li^+^↔ Li_2_S” (the reduction of S_8_ to Li_2_S, i.e., one S_8_ ring hosts 16 Li^+^ ions at the end of discharge, which is a stepwise process involving different lithium poly‐sulfides as intermediate phases). The strong nucleophilic reaction between the polysulfide anions and carbonate electrolyte generally result in the loss of active materials, thereby leading to a rapid capacity fading of batteries through the following reaction (**Scheme** **1**).^[^ ^193^, ^194^ ^]^

Carbonate electrolytes can be divided into two types based on molecular structure: one is linear‐structured carbonate electrolyte (e.g., diethyl carbonate, DEC, dimethyl carbonate, DMC, and ethylene methyl carbonate, EMC) and the other is cyclic‐structured carbonate electrolyte (e.g., ethylene carbonate, EC and propylene carbonate, PC). Both linear and cyclic carbonates react with PS through different reaction mechanisms producing different products. The linear carbonate electrolyte is decomposed by nucleophilic sulfide anions, and they form cascade intermediates like thiocarbonate with methanol/ethanol (in the case of DMC). The formation of CO_2_ can be made possible by the methylation of thiolates (in the case of EMC). Cyclic‐structured carbonate produces an open‐ring intermediate by intramolecular or intramolecular nucleophilic sulfide anions, and then further nucleophilic substitution leads to the formation of thioether. Therefore, carbonate electrolytes are not favorable for the conventional MSBs.^[^ ^195^ ^]^ In contrast, the SPAN cathode overcomes this limitation of Li–S batteries because the sulfur atoms in SPAN are covalently bonded to the carbon backbone of polymer matrix, thereby avoiding the soluble long‐chain PS formation and subsequent dissolution into electrolytes.^[^ ^32^, ^196^ ^]^ Due to the insolubility of the C—S bond and discharge product (M_2_S, M = Li, Na, and K) in organic electrolytes, the SPAN structure is entirely reversible through a solid‐to‐solid phase transition during the charge–discharge process in both carbonate‐ and ether‐based electrolytes.

(3) $$-C-\mathrm{SM}+M^{+}+e^{-}\to -C^{•}+M_{2}S$$

(4) $$-C-\mathrm{SM}+2M^{+}+2e^{-}\to -C^{-}M^{+}+M_{2}S$$

(5) $$-C^{•}+M_{2}S\to -C-\mathrm{SM}+K^{+}+e^{-}$$

Notably, SPAN cathode can be formed in various structures depending on the heat‐treatment condition (see the synthesis and characterization sections), and therefore, the issue of PS formation and dissolution into electrolyte solution remains controversial, necessitating more systematic studies. Considering a high compatibility between SPAN cathode and carbonate electrolyte, the technology transfers of highly developed LIBs into Li–S battery with SPAN cathode is possible. For example, Hwang et al.^[^ ^4^ ^]^ assembled a pouch cell of 100 mAh using the combination of SPAN/PAA cathode with FEC‐containing electrolyte, which exhibited a specific capacity of 970 mAh g^–1^ after 100 cycles at 0.5 C. These authors made a flexible H‐SPAN by electrospinning and assembled a pouch cell, that can deliver a high initial specific capacity of 1700 mAh g^−1^ and maintain at 1100 mAh g^−1^ after 50 cycles.^[^ ^103^ ^]^ Furthermore, Razzaq et al.^[^ ^177^ ^]^ assembled the prototype pouch cell using CoS_2_‐SPAN‐CNT that exhibited a high discharge capacity of 1322 mAh g^−1^. In addition, many researchers have reported that the carbonate‐based electrolyte is more stable than the ether‐based electrolyte in sulfur batteries when using SPAN cathode.^[^ ^32^, ^33^, ^44^, ^75^, ^80^ ^]^ Conversely, the ether‐based electrolyte provides an advantage in stabilizing the metallic anode in MSBs.^[^ ^197^, ^198^ ^]^ In particular, ether solvent with a high donor number and dielectric constant can produce a high concentration of electrolyte. Owing to the unique solvation sheath of metal ions, the concentrated electrolyte can produce stable solid electrolyte interface (SEI) layers and suppress the dissolution of PS and dendritic growth of metallic anode.^[^ ^34^, ^199^, ^200^, ^201^, ^202^, ^203^, ^204^, ^205^, ^206^, ^207^ ^]^ Wang et al.^[^ ^199^ ^]^ recently proposed a new electrolyte made of 4 m LiFSI in dibutyl‐ether (DBE) which can inhibit the dissolution of PS and suppress the formation of Li dendrite. They exhibited better cycling stability and electrochemical reversibility of Li‐SPAN batteries using 4 m LiFSI in DBE.

Undoubtedly, SPAN is an attractive cathode material that has a high compatibility with various electrolyte solutions in MSBs.^[^ ^44^ ^]^ However, studies concerning the effect of electrolyte solutions on SPAN cathode are either in their infancy stage or are scarce. To develop more advanced MSBs using SPAN cathode, simultaneous studies on the effects of various electrolytes on the anode and cathode are necessary.

## 6 Reaction Mechanism of Sulfurized Polyacrylonitrile Electrodes

Although SPAN is a promising electrode for sulfur battery systems towing to its improved stability and lower polysulfide dissolution, its reaction mechanism upon the discharge/charge process has not been fully understood. Wang et al.^[^ ^78^ ^]^ investigated the reaction mechanism against Li, finding that a thiyl radical can be generated after the cleavage of the S—S bond in SPAN structure (Structure I in Figure 7c) in the first cycle. Moreover, it was demonstrated that a conjugative structure formed due to electron delocalization of the thiyl radical on the pyridine backbone. The conjugative structure can react with lithium ions through a lithium‐coupled electron transfer process and form ion‐coordination bond reversibly.

**Figure 16:**
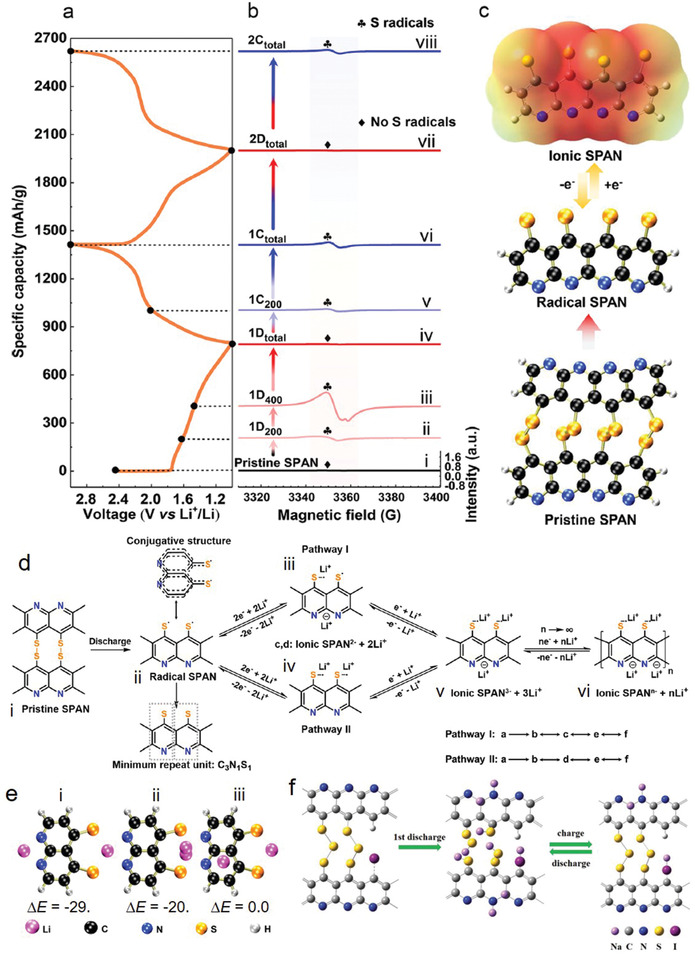
a) Galvanostatic (dis‐)charge curves of SPAN in the first and second cycles, b) corresponding ex situ EPR spectra of SPAN electrode at different discharge and charge states, c) proposed structural evolution from pristine SPAN to the intermediates of radical and ionic SPAN in the reactions, d) schematic of the reaction pathway for SPAN to store Li^+^, i) pristine SPAN. ii) Radical SPAN with a conjugative structure and the corresponding minimum repeat unit. iii,iv) Ionic SPAN^2−^ + 2Li^+^. v) Ionic SPAN^3−^ + 3Li^+^. vi) ionic SPAN*^n^* ^−^ + *n*Li^+^, and e) potential energy of the SPAN radical to host different units of (i–iii) 2 Li^+^ ions in different states. a–e) Reproduced with permission.^[^ ^79^ ^]^ Copyright 2014, Elsevier. f) Proposed reaction mechanism of the I‐S@pPAN during cycling in Na/S battery, Reproducedwith permission. ^65^ ^]^ Copyright 2019, American Chemical Society.

The storage mechanism of Li^+^ in the SPAN structure was further investigated using ex situ electron paramagnetic resonance (EPR) signal from electrodes at different states of charge (**Figure** **16**a,b). In the discharge process, due to reaction between Li^+^ and SPAN, continuous S—S cleavage and the generation of thiyl radicals appeared and gradually increased at the maximum, from pristine SPAN to 1D_400_. The radical intensity weakened at the state of 1D_total_ due to the chemical combination of radicals with Li^+^ following the electron coupling. Reversibly, there was a regeneration of the radical with Li^+^ extraction from the SPAN structure at 1C_total_, maintained in the following charge process. The electron delocalization of conjugative structure on the SPAN structure can be interpreted by the steric configuration of radical and ionic SPAN through simulations, with the possible evolution of the SPAN structure (Figure 16c). From the pristine SPAN, the linear radical SPAN was formed after cleavage of the S—S bond. The linear structure then converted to a “zigzag” shape due to electrostatic repulsion. This kind of molecular structure has a large space and is rich in negative locations, enabling the molecules to have high activity to react with Li^+^.

Based on the above experiments, a reaction mechanism was proposed in Figure 16d. When SPAN accepts electrons from the external circuit, it experiences S—S bond cleavage and reacts with Li^+^ forming lithiated ionic SPAN, and then forms an infinite unit structure of SPAN in the first discharge. The pathway‐i appeared to be preferable due to the low potential energy than those of other two pathways (Figure 16e) and the existence of radicals, as shown in the 1D_200_ and 1D_400_ EPR spectra of Figure 16b. However, the lithiated ionic SPAN can convert to radical SPAN rather than returning to initial SPAN in the charge process. Therefore, the first discharge process starts from the cleavage of the S—S bond in SPAN while all the following discharge processes start from radical SPAN, as evidenced by the dissimilar discharge plateau in the first cycle to that in the subsequent cycles. Furthermore, evidently, the bond formation between Li^+^ and negative locations around sulfur/nitrogen atoms is very rapid, thereby leading to extremely high stability and superior rate capability.

Similarly, Weret et al.^[^ ^77^ ^]^ suggest that the cleavage of not only S—S bond, but also C—S, and N—S bonds of SPAN cathode induce Li addition through Li—S, Li—C, and Li—N bond in the first discharge. In charging state, the S—S and C—S/N—S bonds are reformed releasing Li, suggesting the reversibility of the covalent bond formation. However, the C = N bonds also interact with Li forming irreversible Li—C and Li—N bonds contributing to high initial discharge capacity. The electron‐donating effect of those bonds increases the electron density of the conjugated backbone^[^ ^44^ ^]^ which eventually decrease the charge/discharge voltage hysteresis at further cycle. Jin et al.^[^ ^58^ ^]^ suggests that both C = N and C = C double bonds break and react with Li ions to form Li—C—N—Li and Li—C—C—Li which may not proceed delithiation reaction in the charging state. Therefore, the remained Li ions after first charge are helpful to improve the conductivity and reduce the electrode polarization, so the subsequent discharge potentials are higher than first discharge potential. Although remained Li ions concomitantly leading to the irreversible capacity loss of the first cycle. Similar mechanism has also observed for Na ions when I is doped in SPAN.^[^ ^74^ ^]^ The cleavage of S—S and C—S bonds are observed in I‐S@pPAN cathode when Na ion is introduced to form Na—S, Na—C along with Na—N and Na—I bonds at the first discharge (Figure 16f). The S—S and C—S formation has observed, and Na ions remained connected with N and I after first charge. It is notable that the comparatively higher discharge capacity and cycle stability has could be

## 7 Summary and Prospects

SPAN was demonstrated to have unique properties, such as high sulfur utilization and lower LiPS solubility than sulfur‐based electrodes. However, because of the low sulfur loading in the SPAN structure, debatable chemical structure, and uncertain reaction mechanisms, SPAN‐based batteries experience inferior cycle life and delivered energy density. We reviewed and summarized recent applications of SPAN electrodes against metal anodes, while strongly highlighting how the investigation of its electrochemical mechanism and the design of advanced compound is fundamental in achieving practical applications. A synergistic combination of theoretical calculations and experimental approaches should be attempted in depth. In situ and ex situ characterizations at the nanoscale, upon cycling, are also essential for a comprehensive understanding of the reaction mechanism to clarify controversial and unclear aspects. The optimization of the chemical structure, as well as, the chemical bonding nature is also necessary, together with the utilization of the overall sulfur of SPAN backbone.

Following the concepts described here, inclusion of new functional groups and binder additives to maintain the electric contacts and improve the kinetic of the electrochemical reaction through the design of new 3D structure is a promising way forward to develop SPAN electrodes with high rate capability and practical energy density for future commercial applications. To further enhance the practical acceptability of SPAN cathode, existing challenges and prospects for research are summarized as follows:

### 7.1 Structural Optimization

As described in the introduction, there are primarily three classes of SPAN structures that have been reported so far which resulted in the different atomic ratio of C/S. Thus, the amount of sulfur content in SPAN frequently changes according to the structure, thereby leading to the higher specific capacity than theoretical capacity, lower cycle stability than the expectation, and unclear reaction mechanism because the sulfur in SPAN structure is the most active site. Therefore, it is important to develop advanced characterization techniques, that is, in situ characterizations for the precise determination of SPAN structures.

### 7.2 Increase the Sulfur Loading in Sulfurized Polyacrylonitrile Structure

Most of the SPAN‐based materials have been synthesized by sulfurization under high‐temperature treatment, leading to unpredictable sulfur attachment through covalent bonding and vague SPAN structure. Therefore, the design and preparation through direct chemical reaction is anticipated. Furthermore, the other functional groups can be rationally added or created to improve the battery performance because the nitrogen in SPAN has indirect participation, as observed in the charge/discharge reaction mechanism at the SPAN cathode. For instance, most of the nitrogen atoms in SPAN chain (Figure 3) are bonded with carbon that might be utilized to increase the total sulfur content by bonding with sulfur chain through selective chemical reaction as shown in Figure 4b. For example, S/cPAN/carbon^[^ ^89^ ^]^ contains 51 wt% of sulfur which has introduced by simple chemical activation in the preparation route. Although, several samples have prepared by electrospinning which contain higher wt% of sulfur.^[^ ^18^, ^120^, ^124^ ^]^ The chemical approach is proven further as the efficient way to increase sulfur content.^[^ ^192^ ^]^ In near future, PANI could be another probable candidate in this family to bond sulfur covalently in carbon matrix with increasing sulfur content.

### 7.3 Understanding the Reaction Mechanism

Covalently bonded sulfur active sites in SPAN undergo continuous structural evolution, along with noncovalent sulfur loss during the charge/discharge process, leading to the capacity fading and less durability. Therefore, it is important to fully understand the fundamental mechanisms related to the covalent sulfur conversion reactions by in situ observations.

### 7.4 Research on Electrolytes

Although SPAN cathode has a problem of lower PS dissolution and many conventional electrolytes are suitable for it, the investigation on gel (polymer) electrolyte should be emphasized for better performance because recently the SPAN cathode showed demonstrated a better performance in gel electrolyte. Moreover, research on the finding of brand‐new electrolytes for SPAN cathode as metallic anode should be conducted to form high quality SEI.

### 7.5 Research on Anode Materials

To improve the overall battery performance, investigations should extend to the anode materials. A suitable type of anode material can increase the overall battery performance and cycle stability because metal anode may suffer from several problems such as dendrite formation.

**Table 1:** Summary of various SPAN materials and their performances

			Sulfur			Capacity [mAh $g_{\mathrm{sulfur}}^{-1}$]		
SPAN Materials ^[Ref]^	Synthesis method (PAN:sulfur ratio)	Electrolyte	Content [wt%]	Loading [mg cm^−2^]	C‐rate	Reversible @ cycle	Retention @ cycle	@ Best rate [C]
SPAN^[^ ^4^ ^]^	AN polymerization with AIBN; ball milling; Sulfurization at 350 °C	1 m LiPF_6_ in EC/DEC (1:1)	45	3	0.5	1500^2nd^	98.5%^@100^	≈1050^@3^
SPAN^[^ ^11^ ^]^	Sulfurization at 300 °C; (1:1.2)	1 m LiPF_6_ in EC/DMC (1:1)	53.41	‐	30 mA g^−1^	≈630^2nd^	‐	‐
CNF‐S^[^ ^18^ ^]^	Electrospun at voltage of 15 kV	1 m LiTFSI/0.2 m LiNO_3_ in DOL/DME (1:1)	61.18	4	0.1	≈1000^2nd^	90.3% ^@100^	≈810^@2^
Composite^[^ ^29^ ^]^	Sulfurization at 280–300 °C	1 m LiPF_6_ in EC/DMC (1:1)	53.41	‐	0.2 mA g^−1^	≈880^2nd^	≈600^@50^	‐
Composite^[^ ^30^ ^]^	Sulfurization at 280–300 °C	1 m LiPF_6_ in PC/EC/DEC (1:4:5)	53.41	3.5	0.3 mA g^−1^	≈780^3nd^	≈600^@50^	‐
*c*‐PANS NFs^[^ ^31^ ^]^	Electrospun at 10.5 kV; sulfurization at 450 C; (1:4)	0.8 m NaClO_4_ EC/DEC (1:1)	31.42	≈1.0−1.2	1	≈696^3nd^	≈487^@500^	≈229^@6^
SPAN4^[^ ^33^ ^]^	PAN sulfur ball milling; sulfurization at 450 °C; (1:4)	1 m LiPF_6_ in EC/DEC (1:1)	45.6	‐	0.4	≈1300^3nd^	1000^@1000^	≈1050^@1.6^
SPAN^[^ ^35^ ^]^	AN polymerization with AMPN; Sulfurization at 350 °C; (1:4)	0.5 m KPF_6_ EC/DMC (1:1)	45.5	0.8	0.5	1050^2nd^	997.5^@100^	550^@3^
S/DPAN‐m^[^ ^36^ ^]^	PAN/sulfur mixed manually; sulfurization at 300 °C; (1:4)	1 m LiPF_6_ in EC:DMC:DEC (1:1:1)	48	7	0.2	1343^5th^	74%^@80^	770^@1^
pPAN–S/GNS^[^ ^37^ ^]^	AN polymerization with GO/AIBN; ball‐milled; Sulfurization at 300 °C	1 m LiPF_6_ in EC:DMC (1:1)	48	‐	0.1	1500^2nd^	1200^@100^	800^@6^
SPAN‐NaCMC^[^ ^38^ ^]^	Sulfurization at 350 °C	1 m LiTFSI in DOL/DME (1:1)	32	1	0.9	‐	938^@500^	677^@4.5^
S/DPAN/KB^[^ ^39^ ^]^	Sulfur, KB (95:5) heat at 300 °C; PAN, S/KB (2:3) electrospinning at 17 kV; heating at 300 °C	1 m LiPF_6_ in EC:DMC:DEC (1:1:1)	30.24	1.5	0.1	1128^2nd^	917^@150^	342^@1^
S/DPAN/rGO^[^ ^40^ ^]^	Sulfurization at 300 °C; (1:4); rGO (5%) and S/DPAN was mixed under CTAB; sonication	1 m LiPF_6_ in EC:DMC (3:7)	47	‐	0.2	≈1490^2nd^	92%^@100^	700^@2^
NiS_2_–SPAN^[^ ^41^ ^]^	NiCO_3_, PAN/sulfur (1:3) ball‐milled in EtOH; sulfurization at 350 °C	1 m LiPF_6_ in EC:DMC:DEC (1:1:1)	46.01	1.15	200 mA g^−1^	1722^2nd^	1533^@100^	1180^@2 A/g^
MSPAN^[^ ^42^ ^]^	In situ polymerization with SBA‐15/AIBN; SBA‐15:sulfur (1.8) heated at 330 °C; etching with HF	1 m LiPF_6_ in EC:DEC (1:1)	45.87	2.45	1	≈830^2nd^	755^@200^	350^@5^
S@pPAN‐5^[^ ^43^ ^]^	Ball‐milled; sulfurization at 330 °C under pressure of 5 MPa; (1:3)	1 m LiPF_6_ in EC:DMC:DEC (1:1:1)	45.46	1.9	200 mA g^−1^	1542^2nd^	1357^@100^	1008^@2 A/g^
CSM‐450^[^ ^45^ ^]^	Sulfurization at 450 °C	1 m LiPF_6_ in EC:DMC (1:1)	35.24	4.5	0.2	520^2nd^	480^@240^	‐
SPAN/CNT‐12^[^ ^44^ ^]^	CNTs, PAN in DMF electrospun at 18 kV; PAN/CNT sulfurization at 350 °C	1 m LiTFSI/0.2 M LiNO_3_ in DOL/DME (1:1)	41.02	2	200 mA g^−1^	1390^50th^	1400^@200^	885^@1.6^ ^A/g^
S@pPAN^[^ ^58^ ^]^	PAN/sulfur (1:3) ball‐milled in EtOH; sulfurization at 350 °C	1 m LiPF_6_ in EC:DMC:DEC (1:1:1)	37.64	2.5	200 mA g^−1^	1729^2nd^	1702^@100^	‐
pPAN‐S@MWCNT^[^ ^70^ ^]^	AN, IA, AIBN, MWCNT in DMSO/water (1:1) heat at 65 °C; PAN@MWCNT, sulfur (7:1) heat at 300 °C	1 m LiPF_6_ in EC/DMC (1:1)	48	‐	0.1	697^2nd^	85%^@50^	450^@4^
CSM‐450^[^ ^71^ ^]^	Sulfurization at 450 °C	1 m LiPF_6_ in EC/DMC (1:1)	‐	4.5	0.2 mA cm^−2^	520^2nd^	90%^@380^	‐
SPAN^[^ ^72^ ^]^	Sulfurization at 500 °C (1:3)	1 m LiPF_6_ in EC/EMC (1:1)	‐	0.5	200 mA g^−1^	≈530^2nd^	≈430^@200^	390^@1A/g^
S@pPAN^[^ ^73^ ^]^	Ball‐milled; sulfurization at 350 °C (1:3)	1 m LiPF_6_ in EC/DMC (1:1)	46.60	1	100 mA g^−1^	≈740^2nd^	671^@100^	497^@1A/g^
*c*‐PANS^[^ ^45^ ^]^	Ball‐milled; sulfurization at 450 °C (1:4)	1 m LiTFSI 0.1 m LiNO_3_ 0.05 m CsNO_3_ in DOL/DME (1:1)	39.62	17	0.42 mA cm^−2^	‐	74.1%^@90^	‐
fibrous SPAN^[^ ^34^ ^]^	Heat at 550 °C on PMMA/PAN (1:3) with excess sulfur; Soxhlet extraction	3 m LiTFSI in FEC/DOL (1:1)	43.6	0.672	0.5	1672*	>800^@1200^	380^@8^
SPAN‐B^[^ ^75^ ^]^	Sulfurization at 280–300 °C	1 m LiPF_6_ in EC/DEC	42.0	5#	0.25 mA cm^−2^	700^2nd^	97%^@80^	‐
PS45^[^ ^76^ ^]^	PAN/sulfur aging under N_2_ at RT; Sulfurization at 300 °C (1:1.2)	Li_2_S‐P_2_S_5_ ball‐milled (solid)	‐	‐	26.5 mA g^−1^	659^2nd^	605^@50^	≈450^@2^
SPAN‐SE^[^ ^77^ ^]^	PAN/sulfur ball‐milled in EtOH; sulfurization at 350 °C; Soxhlet extraction (4:1)	1.0 M LiPF_6_ in EC/DEC (1:1)	39.10	0.86	0.1	≈1400^2nd^	1200^@250^	353^@2^
SPAN^[^ ^78^ ^]^	Sulfurization at 330 °C	1.0 m LiPF_6_ in EC/DEC (1:1)	39.79	1.5#	0.025	≈350^5th^	201^@21^	54^@0.5^
Fibrous SPAN^[^ ^80^ ^]^	Heat at 550 °C on PMMA/PAN (1:3) with sulfur; Soxhlet extraction	3 m LiTFSI in FEC/DOL (2:1)	40	‐	0.1	≈900^2nd^	≈790^@50^	‐
Composite^[^ ^86^ ^]^	PVA, AN, sulfur, AIBN in water at 65 °C; Sulfurization at 330 °C	1.0 m LiPF_6_ in EC/DEC (1:1)	33.41	‐	0.1	416.4^2nd^	400^@30^	‐
S/*p*PAN^[^ ^88^ ^]^	AN, PPS, SDS, sulfur in water at 70 °C; Sulfurization at 350 °C	1 m LiPF_6_ in EC/DMC/DEC (1:1:1)	40.9	2#	0.5	1177^2nd^	100^@100^	981^@1^
S/cPAN/carbon^[^ ^89^ ^]^	CB activated KOH; cPAN prepared at 250 °C; cPAN, sulfur in ethanol ball‐milled; sulfurization at 350 °C; S/cPAN, A‐CCB, HCOOH, CTAB mixed; heat at 300 °C; (4:6)	5 m LiTFSI in DOL/DME	51	‐	100 mA g^−1^	≈700^2nd^	493.7^@100^	‐
S/DPAN^[^ ^91^ ^]^	PAN/sulfur ball‐milled; dried at 50 °C; Sulfurization at 300 °C; (1:4)	1 m LiPF_6_ in EC/DMC/DEC (1:1:1)	53.5	10–12#	0.2	1100^3rd^	66%^@50^	‐
PAN–S–VA^[^ ^95^ ^]^	PAN, sulfur, MBT (1:1:0.1) ball‐milled; heat 180 and 280 °C	1 m LiPF_6_ in EC/DMC (1:1)	36.89	‐	0.25	494^2nd^	≈100^@200^	≈280^@2^
Composite^[^ ^96^ ^]^	PAN/sulfur ball‐milled; dried at 60 °C; Sulfurization at 350 °C	1 m LiPF_6_ in EC/DMC/EMC (1:1:1)	‐	‐	100 mA g^−1^	810.5^2nd^	795.4^@50^	‐
SPAN^[^ ^98^ ^]^	Sulfurization at 550 °C; Soxhlet extraction (1:15)	1 m LiPF_6_ in EC/DMC/DEC (2:1:1)	44	5#	0.1	≈1450^2nd^	1429^@37^	‐
SVF^[^ ^102^ ^]^	PAN/PS/VGCF in DMF at 60 °C; ball‐milled; electrospun at 17 kV; PAN/PS/VGCF soaked in CS_2_/S (100:30)	1 m LiPF_6_ in EC/DEC (1:1)	37.78	6.37#	1	955^3nd^	903^@150^	300^@4^
H‐SPAN film^[^ ^103^ ^]^	PEO/PAN core/shell nanofibers by electrospun at 15 kV; ultrasonically remove PEO; H‐PAN/sulfur heated at 390 °C (sealed); sulfurization at 350 °C; (1:3)	1 m LiTFSI in DOL/DME (1:1)	≈42	0.7	0.1	1250^2nd^	1236^@300^	499^@1^
SPAN‐4^[^ ^104^ ^]^	AN, EGDMA, EC/ethanol (1:9), CPDT, AIBN heated to 75 °C; sulfurization at 550 °C with excess sulfur; Soxhlet extraction	3 m LiTFSI in EC/DMC (1:1)	40	0.5	0.25	900^2nd^	95%^@200^	420^@8^
SPAN‐CNTs^[^ ^107^ ^]^	MWCNTs/PAN (1:10) ball‐milled; sulfur in ethanol ball‐milled; sulfurization at 300 °C; (1:10)	1 m LiPF_6_ in EC/DMC (1:1)	35.1	4	0.1	559.6^2nd^	96.5%^@100^	386.7^@7^
SPAN/RGO^[^ ^114^ ^]^	RGO in 5.88 g L^−1^ PAN/DMF (64 mL); add ammonia; PAN/RGO, sulfur ball‐milled; sulfurization at 300 °C; (1:4)	1.0 m LiPF_6_ in EC/DC/DMC (1:1:1)	44	6‐8	0.1	1385^10th^	1100^@200^	828^@2^
pPAN–S@GNS^[^ ^115^ ^]^	GO/AN, PT, SDS in water at 70 °C; GO/PAN deduced at 95 °C; PAN@GNS, sulfur heated at 300 °C; (1:6.4)	1 m LiPF_6_ in EC/DMC (1:1)	47	0.752	0.2	1449.3^2nd^	88.8%^@300^	700^@10^
S/PAN/Graphene^[^ ^116^ ^]^	PAN, Sulfur, graphene (1:4:0.25) ball‐milled; dried at 60 °C; sulfurization at 350 °C;	1 m LiPF_6_ in EC:DMC:DEC (1:1:1)	47.3	3.5	0.1	612^2nd^	77%^@100^	360^@4^
C/S/PAN nanofibers^[^ ^120^ ^]^	BP/sulfur (1:4) heat at 155 °C ball‐milled; add S/C to 8% PAN/DMF solution (5:2); electrospun at 16 kV; sulfurization at 280 °C	1 m LiPF_6_ in PC/EC/DEC (1:4:5)	53	‐	200 mA g^−1^	1183^2nd^	>730^@400^	616^@4^
S‐a‐MCNF^[^ ^46^ ^]^	PAN and PMMA in 10 mL DMF at 60 °C; electrospun at 18 kV; KOH activation; heated at 800 °C. Na_2_S_2_O_3_·5H_2_O and *a*‐MCNF in water; HCl addition; heated 155 °C	1 m LiTFSI/0.4 m LiNO_3_ in DOL/DME (1:1)		2.2	0.2	≈1180^2nd^	920^@300^	847^@5^
CMK‐3/S@PANS@TPS^[^ ^124^ ^]^	CMK‐3 and sulfur (3:7) ball‐milled; heated to 155 °C. CMK‐3/S in water‐DMSO (1:1); added AN and AIBN kept at 65 °C; CMK‐3/S@PAN and sulfur heated at 300 °C; (1:3).	1 m LiTFSI in DOL/DME (1:1) in presence of 2 wt% of TPP	68	2.1	1	994^1st^	698^@1000^	915^@2^
C@S/PAN^[^ ^125^ ^]^	Sulfur, sucrose, PAN (4:1:0.2). PAN in DMF at 90 °C, sucrose and sulfur added after cooling stirring; dried at 80 °C; sulfurization at 450 °C;	1 m LiPF_6_ in EC/DEC (1:1)	‐	2.5	0.1	≈1108^2nd^	1025^@200^	933^@1^
S/PAN/Mg_0.6_Ni_0.4_O^[^ ^130^ ^]^	Mg(NO_3_)_2_, Ni(NO_3_)_2_, glycine in water; boiled, ignited; calcined at 700 °C. Sulfur, PAN, Mg_0.6_Ni_0.4_O (4:1:0.25) ball‐milled; sulfurization at 350 °C.	1 m LiPF_6_ in EC/DMC/DEC (1:1:1)	38.5	4	0.1	1223^2nd^	≈100%^@100^	445^@1^
FeS@SPAN^[^ ^132^ ^]^	PAN and Fe_2_O_3_ in DMF; electrospun at 20 kV; fibers, sulfur sulfurization at 600 °C; (1:6).	1 m LiTFSI in DOL:DME (1:1)	<50	1‐1.2	0.2	1000^2nd^	716^@100^	141.7^@5^
FeS/SPAN‐HNF^[^ ^133^ ^]^	PAN and Fe_2_O_3_ (1:1) dissolved in DMF; ball‐milled; electrospun at 20 kV; Fe_2_O_3_/PAN fiber soaked in 7 wt% S/CS_2_ solution; heated at 600 °C;	1 m NaClO_4_ in EC/PC (1:1) with 5 wt% FEC	38.3	1.5‐2	0.2	782.8^2nd^	≈750^@50^	125.6^@20^
SeS_0.7_/CPAN^[^ ^135^ ^]^	SeS_2_ and PAN mixed (1:1); annealed at 600 °C	1 m LiPF_6_ in EC/DEC (1:1)	‐	1.2	0.6	900^2nd^	780^@1200^	450^@6^
pPAN/SeS_2_^[^ ^136^ ^]^	PAN, PS in DMF electrospun at 17 kV; PAN/PS fibers and SeS_2_ (1:4) heated at 380 °C; heated in furnace at 300 °C.	1 m LiPF_6_ in EC/DEC (1:1)	<50	2	0.5	1020^2nd^	633^@2000^	709^@5^
I‐S@pPAN^[^ ^74^ ^]^	AN, sulfur and iodine (1:3.4:0.6) in ethanol; ball‐milled; dried at 70 °C; sulfurization at 300 °C.	1 m NaClO_4_ in EC/DEC (1:1) + 8% of FEC	42	1	0.1	1261^2nd^	850^@100^	655^@2^
Composite^[^ ^152^ ^]^	PAN/sulfur mixed in EtOH; sulfurization at 300 °C	1 m NaClO_4_ EC/DMC (2:1)	42	‐	‐	530^2nd^	500^@18^	‐
Se_0.08_S_0.92_@*p*PAN^[^ ^153^ ^]^	Sulfur and selenium (18:1) ball‐ milled; heated at 260 °C under vacuum. Se_0.08_S_0.92_, PAN (3:1) annealed at 450 °C.	1.0 m NaClO_4_ in PC/EC (1:1)	36.88	1‐2	0.2	900^2nd^	100%^@200^	767^@3^
Se_0.06_SPAN^[^ ^47^ ^]^	Sulfur and selenium (12:1) ball‐ milled; autoclaved at 250 °C. Se_0.06_S, PAN (3:1) ball‐milled; annealed at 300 °C.	1 m LiTFSI/2% LiNO_3_ in DME/DOL (1:1)	47.25	1‐3	0.26	1230^2nd^	881^@800^	900^@6.5^

*Capacity based on sulfur; #total material loading; AIBN: azodiisobutyronitrile; AMPN: 2,2′‐Azobis(2‐methylpropionitrile); rGO: reduced graphene oxide; CTAB: cetyltrimethylammonium bromide; DMF: N,N‐dimethylformamide; IA: itaconic acid; DMSO: dimethyl sulfoxide; RT: room temperature; PVA: polyvinyl alcohol; PPS: potassium persulfate; SDS: sodium dodecyl sulfate; MBT: 2‐mercaptobenzothiazoles, VGCFs: vapor grown carbon fibers; BP: black pearls 2000; PEO: polyethylene oxide; PT: potassium thiosulfate; Conductive agent:—AB: acetylene black; KB: Ketjen black, CB: Carbon black; DB: denka black; Binder:—NaCMC: sodium carboxymethyl cellulose; SBR/CMC: styrene‐butadiene rubber/carboxymethyl cellulose; PAA: polyacrylic acid; PTFE: polytetrafluorethylene; CMC: carboxymethyl cellulose; Electrolytes:—LiTFSI: lithium bistrifluoromethanesulfonimidate [LiN(SO_2_CF_3_)_2_]; FEC: fluoroethylene carbonate; DOL: 1,3‐dioxolane; LiPF_6_: lithium hexafluorophosphate; KPF_6_: potassium hexafluorophosphate.

## Conflict of Interest

The authors declare no conflict of interest.
